# Hepatic CBP/p300 Orchestrate Amino Acid‐Driven Gluconeogenesis through Histone Crotonylation

**DOI:** 10.1002/advs.202507635

**Published:** 2025-08-12

**Authors:** Chunxiang Sheng, Tianjiao Li, Hong Lin, Xiaoqin Ma, Feiye Zhou, Mingzhu Li, Yiru Wang, Shushu Wang, Jialin Tan, Junmin Chen, Yulin Yang, Jianmin Liu, Yufang Bi, Jieli Lu, Xiao Wang, Libin Zhou

**Affiliations:** ^1^ Department of Endocrine and Metabolic Diseases Shanghai Institute of Endocrine and Metabolic Diseases Ruijin Hospital Shanghai Jiao Tong University School of Medicine Shanghai 200025 China; ^2^ Shanghai National Clinical Research Center for Metabolic Diseases Key Laboratory for Endocrine and Metabolic Diseases of the National Health Commission of the PR China Shanghai National Center for Translational Medicine Ruijin Hospital Shanghai Jiao Tong University School of Medicine Shanghai 200025 China; ^3^ Department of Endocrine and Metabolic Diseases Renji Hospital Shanghai Jiao Tong University School of Medicine Shanghai 200127 China; ^4^ Department of VIP Clinic Shanghai East Hospital Tongji University School of Medicine Shanghai 200120 China; ^5^ Academy of integrative Medicine Shanghai University of Traditional Chinese Medicine Shanghai 201203 China

**Keywords:** 2‐aminoadipic acid, amino acids, CBP/p300, GCDH, gluconeogenesis, histone crotonylation, type 2 diabetes

## Abstract

The role of amino acid metabolism dysregulation in the development of type 2 diabetes remains elusive. Here, significant associations of human *CREBBP/EP300* gene polymorphisms with circulating amino acid and glucose levels are reported. Through integrated transcriptomic, metabolomic, and CUT&Tag analyses, the molecular mechanisms underlying these correlations are investigated. Liver‐specific *Crebbp*/*Ep300* double knockout mice display elevated plasma amino acid levels and impaired hepatic glucose production caused by the downregulation of amino acid metabolism genes, which is closely linked to altered histone crotonylation and acetylation patterns at their promoters. However, key gluconeogenic genes *Pck1* and *G6pc* are not downregulated in knockout mice. Interestingly, the level of 2‐aminoadipic acid (2‐AAA), a biomarker of diabetes, is significantly increased due to decreased glutaryl‐CoA dehydrogenase (GCDH) expression in CBP/p300‐deficient livers. Treatment with 2‐AAA or overexpression of GCDH enhances amino acid‐driven gluconeogenesis through histone crotonylation‐mediated transcriptional activation of amino acid metabolism genes in primary mouse hepatocytes, whereas GCDH knockdown exhibits an opposite result. Furthermore, targeted hepatic knockdown of CBP/p300 markedly attenuates hepatic glucose production from amino acids in diabetic mice. In sum, these findings underscore the pivotal role of CBP/p300 in linking amino acid catabolism to gluconeogenesis through histone crotonylation in a cell‐autonomous manner.

## Graphical abstract

Hepatic CBP/p300 control amino acid‐driven gluconeogenesis by modulating histone crotonylation. This study identifies 2‐aminoadipate (2‐AAA) as a key metabolite that enhances crotonylation and activates amino acid and gluconeogenic gene expression. The CBP/p300‐2‐AAA‐crotonylation axis reveals a nutrient‐sensing epigenetic mechanism and offers a potential therapeutic target for metabolic imbalance.

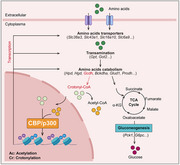

## 1 Introduction

Amino acids function not only as protein synthesis substrates but also as signaling molecules that regulate cellular metabolism and growth, providing intermediates for the tricarboxylic acid (TCA) cycle and gluconeogenesis.^[^ ^1^ ^]^ Cellular and systemic amino acid homeostasis is tightly regulated to maintain physiological health.^[^ ^2^ ^]^ Dysregulated amino acid metabolism has been implicated in a number of pathological conditions, including metabolic disorders, cardiovascular diseases, immune dysfunction, and various cancers.^[^ ^3^ ^]^ Large‐scale human metabolomic studies have provided compelling evidence that elevated circulating branched‐chain amino acids (BCAAs) and aromatic amino acids (AAAs) are strongly associated with increased risk of type 2 diabetes.^[^ ^4^, ^5^, ^6^, ^7^, ^8^ ^]^ Other amino acids, such as alanine, glutamate, aspartate, and lysine, have also been linked to the development of type 2 diabetes,^[^ ^9^, ^10^, ^11^ ^]^ suggesting that increased amino acid availability contributes to disrupted glucose metabolism. It is well‐known that glucogenic amino acids such as alanine and glutamine can drive gluconeogenesis directly by serving as substrates and indirectly by modulating the secretion of glucoregulatory hormones.^[^ ^12^, ^13^, ^14^, ^15^ ^]^ In contrast, certain ketogenic amino acids like leucine and lysine can neither provide substrates for gluconeogenesis, nor elicit glucagon secretion from pancreatic α cells.^[^ ^16^, ^17^ ^]^ However, the catabolite of lysine 2‐aminoadipic acid (2‐AAA) shows a strong correlation with the incidence of type 2 diabetes.^[^ ^7^, ^18^, ^19^ ^]^ The molecular mechanisms underlying this association need to be further explored.

It has been demonstrated that many metabolites function as critical signaling molecules that orchestrate cellular activities in response to nutrient availability.^[^ ^20^ ^]^ Over the past decade, the relationship between histone lysine acetylation and gene expressions has been firmly established.^[^ ^21^ ^]^ More recently, diverse acyl modifications dependent on acyl‐CoAs as donors have been identified on histone lysine residues (e.g., propionylation, butyrylation, glutarylation, crotonylation, and β‐hydroxybutyrylation).^[^ ^22^, ^23^, ^24^, ^25^ ^]^ These acyl‐CoAs are generated through various metabolic pathways, including amino acid catabolism.^[^ ^26^ ^]^ Lysine or tryptophan catabolism generates crotonyl‐CoA, which regulates histone crotonylation levels. Recent studies have demonstrated a pivotal role for histone crotonylation in gene regulation,^[^ ^27^ ^]^ with lysine catabolism shown to reprogram tumor immunity through this modification.^[^ ^28^ ^]^ The diabetes biomarker 2‐AAA undergoes stepwise metabolic conversion and is catalyzed into crotonyl‐CoA by glutaryl‐CoA dehydrogenase (GCDH).^[^ ^29^ ^]^ Whether the intermediates of amino acid metabolism regulate glucose homeostasis through histone acyl modifications is deserved to be investigated.

Apart from specific substrates, the regulation of histone acyl modifications involves a dynamic balance between the enzymatic activities of writers and erasers. CREB‐binding protein (CBP) and p300 constitute one of the three major families of histone acetyltransferases (HATs) that utilize various acyl‐CoAs as substrates for histone lysine acylation.^[^ ^22^, ^30^, ^31^ ^]^ Furthermore, CBP/p300 have been identified as key regulators of energy homeostasis.^[^ ^32^ ^]^ Genome‐wide association study (GWAS) and network analyses have identified CBP as the most interconnected gene in protein‐protein interactions in type 2 diabetes.^[^ ^33^ ^]^ In our study population from the China Cardiometabolic and Cancer Cohort (4C), single nucleotide polymorphisms (SNPs) in the *CREBBP* (encoding CBP) and *EP300* (encoding p300) showed strong associations with circulating amino acid and glucose levels. Therefore, whether CBP/p300 coordinate cellular metabolism by modulating histone acyl modifications in response to amino acids fluctuations warrants further investigation.

The liver is a central hub of amino acid and glucose metabolism and dynamically adapts to nutrient availability.^[^ ^34^ ^]^ Given that CBP and p300 are widely recognized as functionally redundant proteins,^[^ ^35^ ^]^ for the first time we generated hepatocyte‐specific *Crebbp/Ep300* double knockout (CBP/p300^LivDKO^) mice by crossing *Crebbp^flox/flox^/Ep300^flox/flox^* mice with *Albumin* (*Alb*)‐enhancer/promoter driven‐*Cre* transgenic mice to elucidate the role of CBP/p300 in the maintaining amino acid and glucose homeostasis. The knockout mice exhibited elevated plasma amino acid levels due to the reprogramming of hepatic amino acid metabolism, resulting in impaired amino acid‐driven gluconeogenesis without decreasing *Pck1* and *G6pc* expressions. We further explored the molecular mechanisms by which CBP/p300 link amino acid catabolism to glucose homeostasis, providing insights into the development of therapeutic strategies for type 2 diabetes.

## 2 Results

### 2.1 Association of *CREBBP/EP300* SNPs with Circulating Amino Acid and Glucose Levels

**Figure 1:**
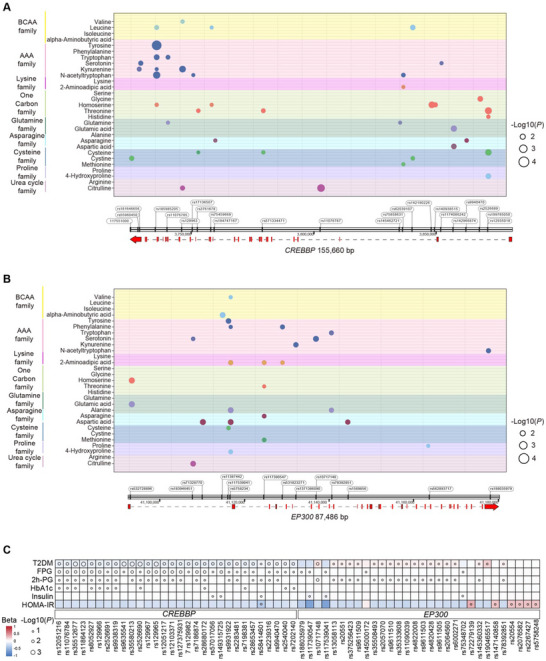
Association of *CREBBP/EP300* SNPs with circulating amino acid levels. A,B) SNPs within *CREBBP* and *EP300* loci exhibit significant correlations with circulating amino acids levels among Chinese adults. The SNP positions are depicted as colored circles, with circle size proportional to statistical significance (−log 10(*p*‐value)). Different colors on the left of the *y*‐axis distinguish amino acid families. The *x*‐axis shows physical positions of each SNP spanning the two genes. C) Correlations between SNPs in *CREBBP/EP300* gene loci and key clinical parameters of type 2 diabetes. The size of each circle is proportional to the significance level, and the shading in each square corresponds proportionally to the beta coefficient.

CBP and its paralog p300 function as histone acetyltransferases and transcriptional coactivators, critically involved in modulating chromatin structure and orchestrating transcriptional programs essential for metabolic homeostasis.^[^ ^32^ ^]^ SNPs within the *CREBBP/EP300* genes can alter protein function and expression levels, thereby affecting metabolic pathways and disease susceptibility.^[^ ^36^, ^37^, ^38^ ^]^ Here, we investigated the associations between SNPs in human *CREBBP/EP300* gene loci and circulating amino acid profiles in our 4C study population.^[^ ^7^ ^]^ We identified 78 SNPs within the *CREBBP* gene locus and 36 SNPs within the *EP300* gene locus (Table S1, Supporting Information). Gene association analysis revealed significant correlations between 23 specific *CREBBP* SNPs and various circulating amino acids, including BCAAs and AAAs (**Figure** **1**A). Similarly, 14 *EP300* SNPs were closely associated with circulating amino acid profiles (Figure 1B). Furthermore, genetic variants in *CREBBP/EP300* genes were associated with key clinical parameters of glucose homeostasis (Figure 1C). These findings suggest a potential role of CBP/p300 in linking amino acid metabolism to glucose homeostasis.

### 2.2 Hepatic *Crebbp* and *Ep300* Double Knockout Mice Exhibit Elevated Levels of Plasma Amino Acids

**Figure 2:**
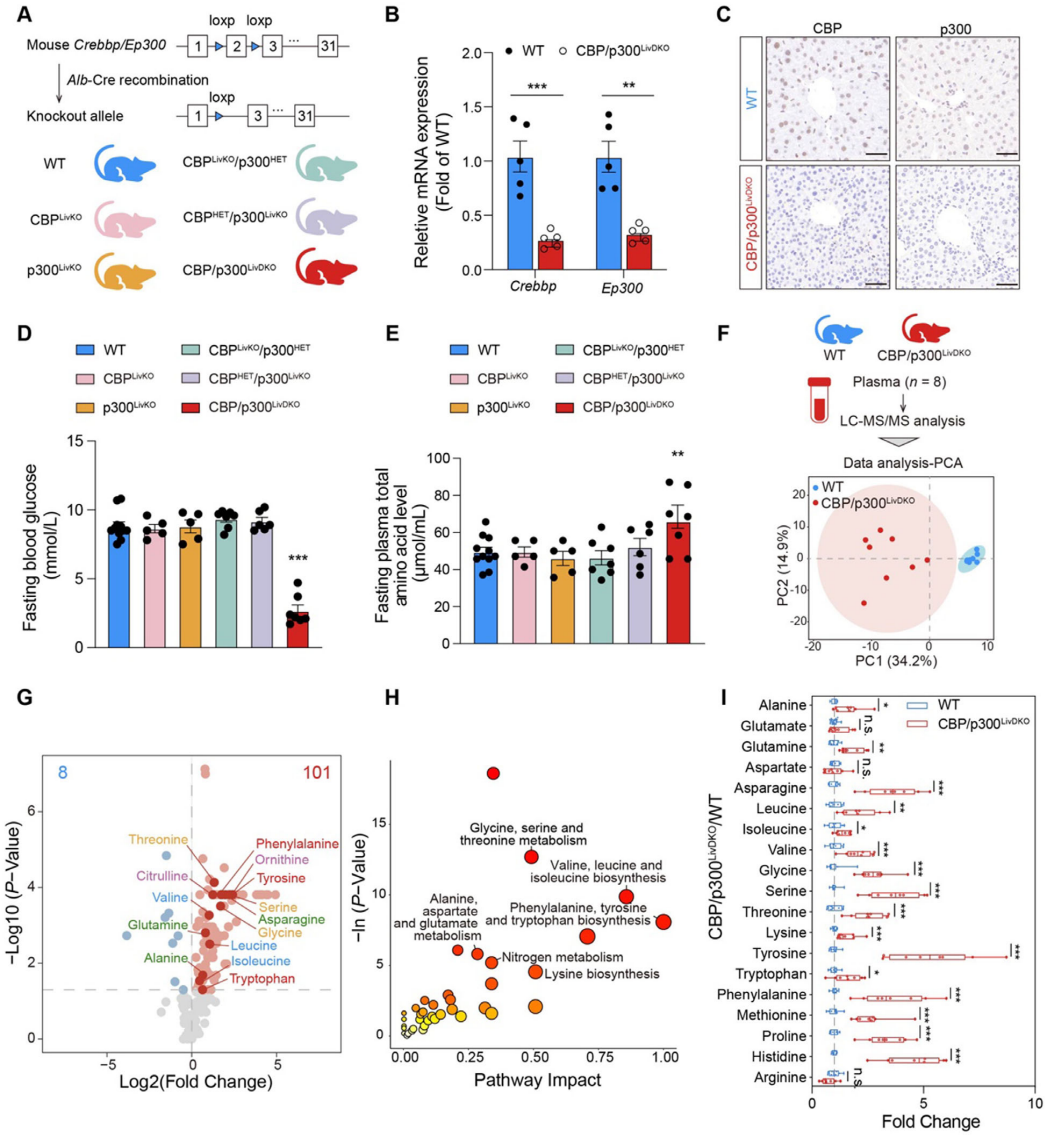
Hepatic *Crebbp* and *Ep300* double knockout mice exhibit elevated levels of plasma amino acids. A) Schematic diagram illustrating the generation of liver‐specific *Crebbp* and *Ep300* knockout (CBP/p300^LivDKO^) mice. B) Hepatic *Crebbp* and *Ep300* mRNA expressions from WT and CBP/p300^LivDKO^ mice (*n* = 5). C) Immunohistochemistry staining of CBP and p300 in the livers of WT and CBP/p300^LivDKO^ mice. Scale bar = 100 µm. D,E) Blood glucose and plasma total amino acid levels in 6‐h fasted mice from six groups: WT (*n* = 11), CBP^LivKO^ (*n* = 5), p300^LivKO^ (*n* = 5), CBP^LivKO^/p300^HET^ (*n* = 7), CBP^HET^/p300^LivKO^ (*n* = 6), CBP/p300^LivDKO^ (*n* = 7). F) Schematic workflow for sample collection and targeted metabolomics profiling of plasma from 6‐h fasted WT and CBP/p300^LivDKO^ mice (*n* = 8). A PCA score plot illustrating distinct metabolic signatures in the two groups. G) Volcano plot showing metabolite abundance discrepancies between WT and CBP/p300^LivDKO^ mice. Significantly differentially abundant metabolites are represented in red (*n* = 101) and blue (*n* = 8). The horizontal line indicates the significance cut‐off of *p* < 0.05. H) Known metabolic pathways involved in amino acid metabolism are enriched among the plasma metabolites in WT and CBP/p300^LivDKO^ mice. The circle colors indicate the level of enrichment significance, with red indicating high significance and yellow indicating low significance. Additionally, larger bubble radii indicate greater pathway impact (Hypergeometric test; *p* < 0.05). I) Interleaved box and whiskers plot showing amino acid levels in WT and CBP/p300^LivDKO^ mice (*n* = 8). Data are presented as mean ± SEM. Statistical significance was determined using two‐tailed unpaired Student's *t*‐test (B,I), or Mann–Whitney *U* test (I) based on data distribution, or one‐way ANOVA followed by Fisher's LSD test (D,E), compared with WT group: ^*^ *p* < 0.05, ^**^ *p* < 0.01, and ^***^ *p* < 0.001; N.S., not significant.

Given the liver's unique role in controlling amino acid and glucose metabolism, we generated hepatic‐specific *Crebbp/Ep300* knockout mouse strains: wild‐type (WT), CBP^LivKO^, p300^LivKO^, CBP^LivKO^/p300^HET^, CBP^HET^/p300^LivKO^, and CBP/p300^LivDKO^ by crossbreeding *Crebbp^flox/flox^* and *Ep300^flox/flox^* mice (with loxP sites flanking exon 2) with *Alb*‐Cre transgenic mice (**Figure** **2**A). Genomic PCR analysis confirmed the indicated genotypes (Figure S1A, Supporting Information). Targeted knockout of *Crebbp* and *Ep300* in the liver was validated by quantitative real‐time PCR (RT‐qPCR) and immunohistochemistry (IHC) (Figure 2B,C), while their expressions were preserved in non‐hepatic tissues (Figure S1B, Supporting Information). Hepatocytes isolated from CBP/p300^LivDKO^ mice also exhibited dramatic decreases in both genes at mRNA and protein levels compared to WT hepatocytes (Figure S1C,D, Supporting Information).

We observed a robust decrease in fasting blood glucose level in CBP/p300^LivDKO^ mice, while no differences were found in other knockout strains compared to WT controls (Figure 2D). Concurrently, plasma levels of triglycerides (TG), non‐esterified fatty acids (NEFAs), and total cholesterol (TC) remained unchanged across all groups (Figure S1E–G, Supporting Information). Given the observed association between *CREBBP/EP300* genetic variations and circulating amino acid profiles in humans, we assessed fasting plasma total amino acid levels across all six mouse groups. Only CBP/p300^LivDKO^ mice exhibited markedly elevated levels of plasma total amino acid compared to WT controls (Figure 2E). These results validate the redundant functions of CBP and p300 in glucose and amino acid homeostasis.

To comprehensively characterize metabolic differences between WT and CBP/p300^LivDKO^ mice, we performed liquid chromatography‐mass spectrometry (LC‐MS)‐based metabolomics to quantify plasma metabolite abundance. Principal‐component analysis (PCA) revealed a clear separation between WT and knockout mice (Figure 2F). Among 189 identified metabolites, differentially expressed metabolites (DEMs) were primarily categorized into organic acids and amino acids (Figure S1H, Supporting Information). Univariate statistical analysis (*p* < 0.05) identified 101 upregulated and 8 downregulated metabolites in CBP/p300^LivDKO^ mice. BCAAs (valine, leucine, and isoleucine), AAAs (tyrosine, phenylalanine, and tryptophan), alanine and glutamine, and glycine were found to be significantly elevated in the plasma of knockout mice. Additionally, the levels of citrulline and ornithine, essential components of the urea cycle, were increased in CBP/p300^LivDKO^ mice (Figure 2G). Metabolic pathway enrichment analysis revealed that altered metabolites were primarily associated with amino acid metabolism, including glycine, serine, and threonine metabolism; valine, leucine, and isoleucine biosynthesis; phenylalanine, tyrosine, and tryptophan biosynthesis; as well as alanine, aspartate, and glutamate metabolism (Figure 2H and Figure S1I, Supporting Information). Comprehensive analysis integrating DEMs through both univariate and multivariate statistical methods (VIP ≥ 1) identified 81 potential biomarkers, including 31 amino acids and derivatives (Figure S1J, Supporting Information). Among the 20 basic amino acids examined, 17 showed significant increases besides glutamate, aspartate, and arginine (Figure 2I). These findings suggest that hepatic CBP/p300 is essential for maintaining systemic amino acid homeostasis.

### 2.3 Amino Acid Metabolic Reprogramming in the Liver of CBP/p300^LivDKO^ Mice

**Figure 3:**
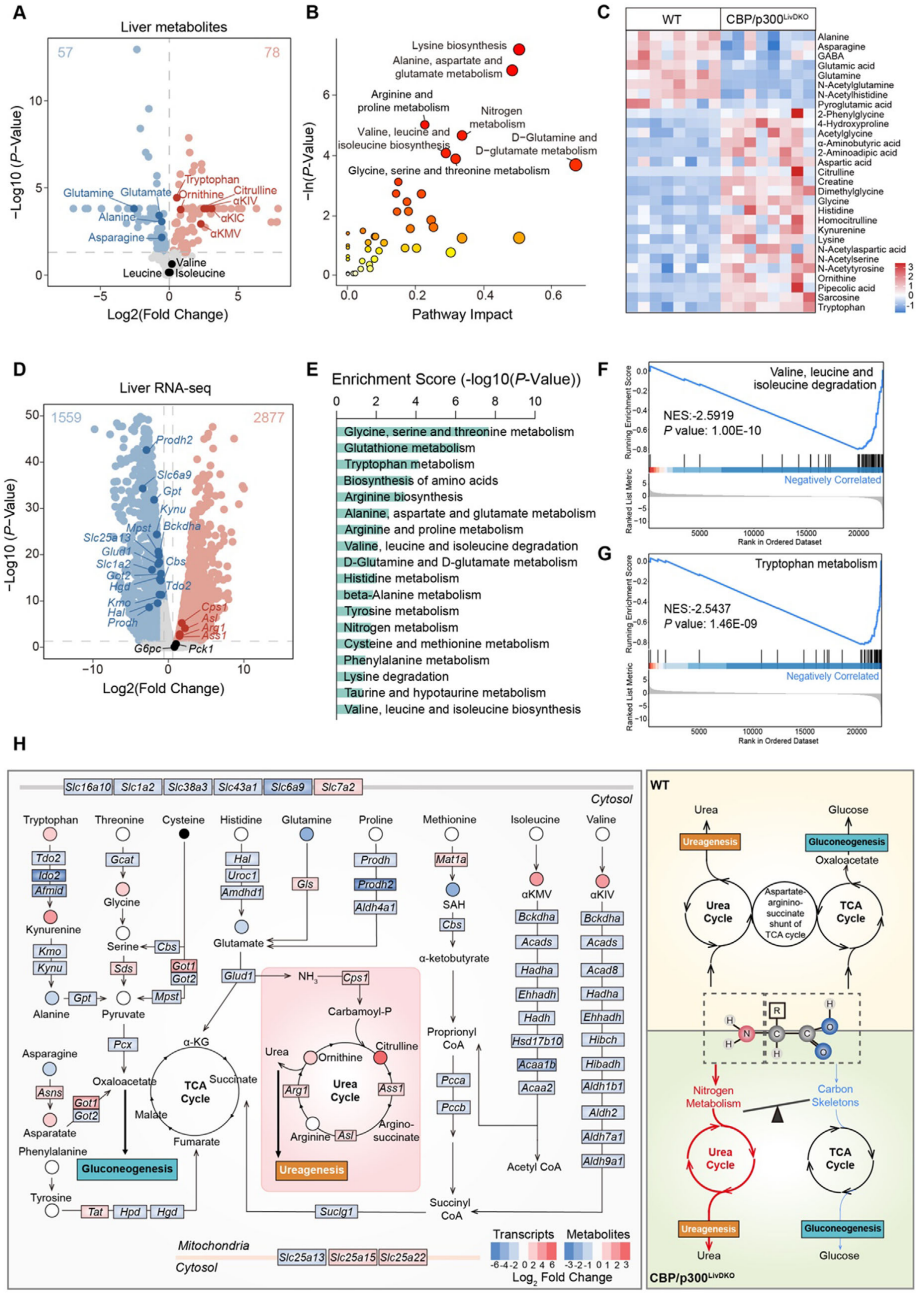
Amino acid metabolic reprogramming in the liver of CBP/p300^LivDKO^ mice. A) Volcano plot depicting differential hepatic metabolites in 6‐h fasted WT and CBP/p300^LivDKO^ mice (*n* = 8). Red (*n* = 78) and blue (*n* = 57) indicate significantly up‐ and down‐regulated metabolites, respectively. B) Bubble plot illustrates pathway enrichment analysis using significantly altered metabolites (Hypergeometric test; *p* < 0.05). C) Heatmap of significant changes in amino acid metabolites in the livers of WT and CBP/p300^LivDKO^ mice. Rows represent the *Z* scores calculated for each group. D) Volcano plot showing differentially expressed genes in the livers of 6‐h fasted WT and CBP/p300^LivDKO^ mice (*n* = 3). The two vertical lines denote cutoff points for a 1.5‐fold change, while the horizontal line indicates the significance cut‐off of *p* < 0.05. E) KEGG Pathway enrichment analysis of RNA‐seq data from the livers of WT and CBP/p300^LivDKO^ mice. Pathways are clustered with similar colors based on their overall function. F,G) GSEA plots demonstrating the valine, leucine, and isoleucine degradation pathway and tryptophan metabolism pathway in RNA‐seq data. NES, normalized enrichment score. H) Integration map of metabolomic and transcriptomic analysis related to amino acid metabolism. Circles represent metabolites detected by LC‐MS/MS (*n* = 8), while rectangles represent transcripts from RNA‐seq data (*n* = 3). Integration based on KEGG pathway annotations.

To determine whether altered plasma amino acid profiles resulted from disrupted hepatic amino acid metabolism in knockout mice, we performed metabolomic analysis on eight liver samples per group, identifying 213 metabolites. PCA revealed a clear separation between datasets, indicating distinct metabolic profiles between the two groups (Figure S2A, Supporting Information). We observed significant metabolic alterations (*p* < 0.05), with 78 metabolites upregulated and 57 downregulated in CBP/p300^LivDKO^ mice (**Figure** **3**A). Consistent with the plasma changes, CBP/p300 knockout resulted in elevated hepatic levels of tryptophan, ornithine, and citrulline. Inconsistent with unchanged levels of plasma branched‐chain keto acids (BCKAs), these BCAA metabolic byproducts increased in the livers of CBP/p300^LivDKO^ mice, while BCAA concentrations remained comparable to those of WT controls. Metabolic pathway enrichment analysis of hepatic DEMs revealed predominant alterations in amino acid metabolism pathways, notably lysine biosynthesis as well as alanine, aspartate, and glutamate metabolism (Figure 3B). Differentially expressed amino acids and derivatives highlighted significant changes in these metabolic pathways (Figure 3C).

To decipher the complex metabolic phenotype of the CBP/p300^LivDKO^ mice, we performed RNA sequencing to explore the role of CBP/p300 in the control of liver metabolism, identifying 2877 upregulated and 1559 downregulated genes between knockout and WT liver samples (|fold change| >1.5 and *p* < 0.05). We observed downregulation of diverse amino acid metabolism genes in the livers of the knockout mice, including amino acid catabolism (*Prodh*, *Prodh2*, *Kynu*, *Bckdha*, *Mpst*, *Glud1*, *Cbs*, *Tdo2*, *Hgd*, *Kmo*, and *Hal*), transamination (*Gpt* and *Got2*), and amino acid transporters (*Slc6a9*, *Slc1a2*, and *Slc25a13*). Conversely, ureagenesis‐related genes (*Cps1*, *Asl*, *Arg1*, and *Ass1*) were significantly upregulated in CBP/p300^LivDKO^ mice. Strikingly, the expression levels of gluconeogenic genes *Pck1* and *G6pc*, previously reported as downstream targets of CBP/p300,^[^ ^39^, ^40^ ^]^ remained comparable between genotypes (Figure 3D). Consistent with previous studies,^[^ ^41^ ^]^ KEGG pathway enrichment analysis revealed significant impacts of hepatic CBP/p300 deletion on glucose and lipid metabolism, indicated by prominent purple and blue bars, respectively (Figure S2B, Supporting Information). Multiple amino acid metabolism pathways were enriched, including glycine, serine, threonine, tryptophan, arginine, alanine, aspartate, glutamate, BCAAs, histidine, tyrosine, lysine, urea cycle metabolism (green bars) (Figure 3E). Gene set enrichment analysis (GSEA) revealed downregulation of genes related to valine, leucine, and isoleucine degradation (Figure 3F), tryptophan metabolism pathway (Figure 3G), and tyrosine metabolism pathway (Figure S2C, Supporting Information) in knockout mice. These findings underscore the pivotal role of CBP/p300 in hepatic amino acid metabolism.

Integrating transcriptomic data with metabolite profiling provided a comprehensive overview of hepatic amino acid metabolic reprogramming in CBP/p300^LivDKO^ mice. Widespread alterations were uncovered in amino acid transporters (including *Slc16a10*, *Slc7a2*, *Slc38a3*, *Slc43a1*, and *Slc6a9*) and numerous catabolic enzymes across multiple amino acid pathways, particularly affecting tryptophan, threonine, histidine, glutamine, and BCAA metabolism. These transcriptional changes were mirrored by corresponding metabolite level shifts, indicating profound metabolic rewiring (Figure 3H). Within the broader context of amino acid metabolism disruption, we observed paradoxical upregulation of the urea cycle alongside downregulation of amino acid carbon skeleton metabolism. Elevated plasma levels of urea cycle intermediates ornithine and citrulline, as well as urea, were observed in CBP/p300^LivKO^ mice (Figure S2D,E, Supporting Information). Additionally, a decreased plasma arginine: (ornithine + citrulline) ratio^[^ ^42^ ^]^ indicated hyperactivation of urea cycle in knockout mice (Figure S2F, Supporting Information). Consistently, hepatic levels of ornithine and citrulline were also elevated in CBP/p300^LivDKO^ mice (Figure S2G, Supporting Information). Ureagenesis was significantly increased in hepatocytes isolated from these mice (Figure S2H, Supporting Information), accompanied by enhanced expression of key urea cycle enzymes (Figure S2I,J, Supporting Information). These results suggest that hepatic double knockout of CBP/p300 induces amino acid metabolic reprogramming, highlighting distinctive decoupling between amino acid carbon skeletons and nitrogen metabolism.

### 2.4 Impaired Amino Acid‐Driven Gluconeogenesis in CBP/p300^LivDKO^ Mice

**Figure 4:**
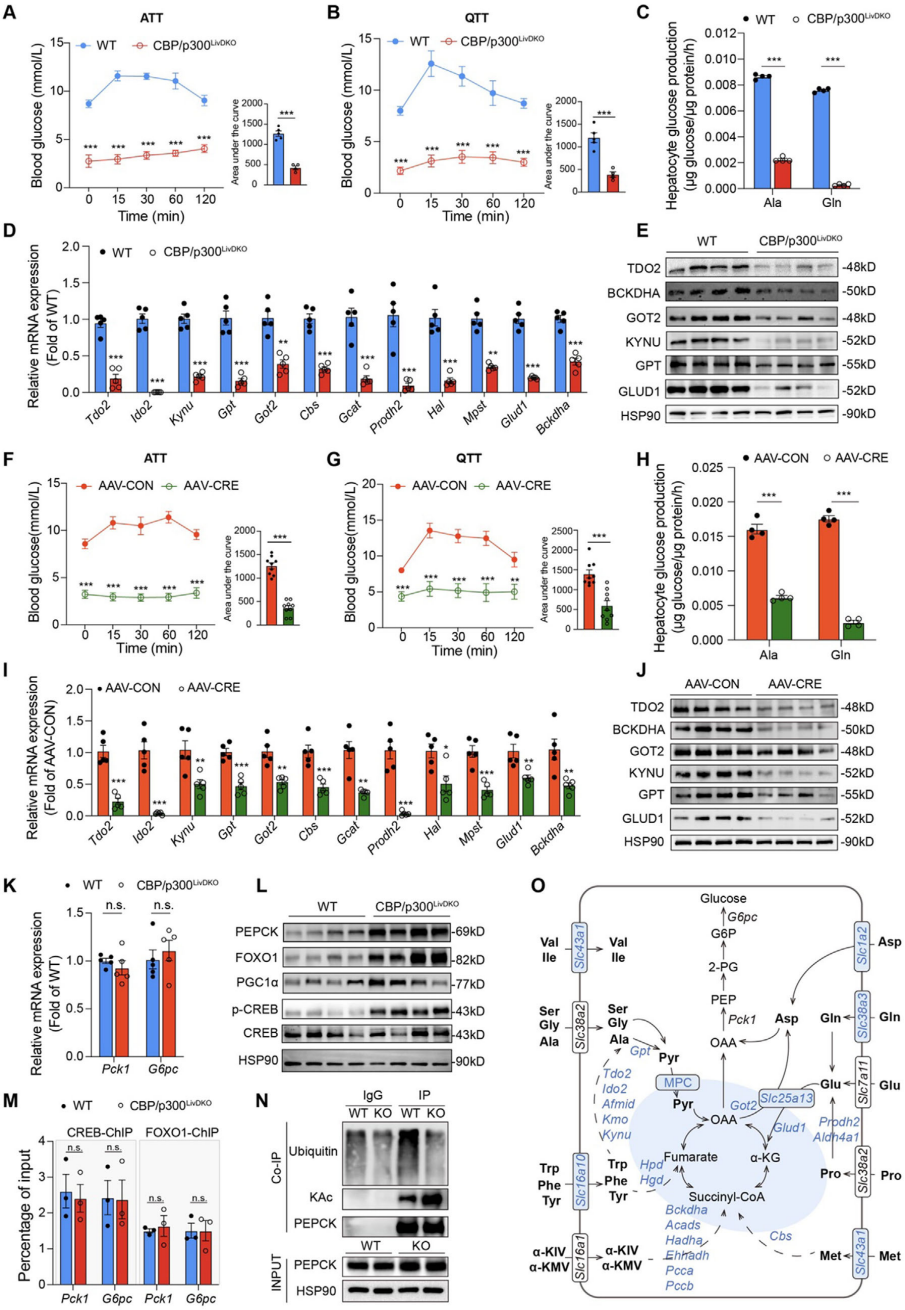
CBP/p300^LivDKO^ mice display severely impaired amino acid‐driven gluconeogenesis. A,B) Blood glucose levels of alanine or glutamine tolerance tests comparing WT (*n* = 5) and CBP/p300^LivDKO^ mice (*n* = 4). Area under the curve was calculated and compared using two‐tailed unpaired Student's *t*‐test. C) Amino acid‐driven gluconeogenesis by primary hepatocytes isolated from WT and CBP/p300^LivDKO^ mice, supported by 10 m L‐alanine or 10 mMm L‐glutamine (*n* = 4). D,E) Hepatic mRNA (*n* = 5) and protein (*n* = 4) expressions of genes related to amino acid metabolism in 6‐h fasted WT and CBP/p300^LivDKO^ mice. F,G) Blood glucose levels of alanine or glutamine tolerance tests comparing AAV‐CON (*n* = 9) and AAV‐CRE mice (*n* = 9). The area under the curve was calculated. H) Amino acid‐driven gluconeogenesis by primary hepatocytes isolated from AAV‐CON and AAV‐CRE mice, supported by 10 mM L‐alanine or 10 mM L‐glutamine (*n* = 4). I,J) Hepatic mRNA (*n* = 5) and protein (*n* = 4) expressions of genes related to amino acid metabolism from 6‐h fasted AAV‐CON and AAV‐CRE mice. K) RT‐qPCR analysis of hepatic mRNA expression of *Pck1* and *G6pc* in WT and CBP/p30^LivDKO^ mice (*n* = 5). L) Western blot analysis of gluconeogenesis‐related proteins in WT and CBP/p300^LivDKO^ mice (*n* = 4). M) ChIP‐qPCR analysis of gluconeogenic gene promoters using antibodies against CREB and FOXO1 in the livers from 6‐h fasted WT and CBP/p300^LivDKO^ mice (*n* = 3). N) Endogenous PEPCK protein was precipitated in the livers of WT and CBP/p300^LivDKO^ mice, and its acetylation and ubiquitination were detected. O) Scheme illustrating the amino acid uptake and catabolism pathways for gluconeogenesis. Genes significantly downregulated in the livers of CBP/p300^LivDKO^ mice are shown in blue. Data are presented as mean ± SEM. Statistical significance was determined using two‐tailed unpaired Student's *t*‐test, compared with WT or AAV‐CON group, ^*^ *p* < 0.05, ^**^ *p* < 0.01, and ^***^ *p* < 0.001; N.S., not significant.

The disruption of hepatic amino acid catabolism prompted us to investigate the capacity of the liver to produce glucose from amino acids in CBP/p300^LivDKO^ mice. Among the 15 gluconeogenic amino acids, alanine and glutamine play dominant roles in gluconeogenesis. Therefore, we conducted alanine tolerance test (ATT) and glutamine tolerance test (QTT) to assess hepatic glucose production in 6‐h fasted mice. Intraperitoneal injection of alanine or glutamine significantly elevated blood glucose levels in WT mice, but not in CBP/p300^LivDKO^ mice (**Figure** **4**A,B). We further performed gluconeogenesis assays using isolated primary hepatocytes treated with alanine and glutamine. Hepatocytes from CBP/p300‐deficient mice showed markedly reduced efficiency in glucose production from these gluconeogenic amino acids compared to WT mice (Figure 4C), accompanied by downregulated mRNA and protein levels of several amino acid catabolism genes (Figure 4D,E).

To further determine whether CBP/p300 is required for hepatic amino acid‐driven gluconeogenesis, we injected *Crebbp^flox/flox^*/*Ep300^flox/flox^* mice with adeno‐associated virus expressing Cre recombinase under a liver‐specific promoter (AAV‐CRE). Mice were tested 3 weeks post AAV‐CRE injection (Figure S3A, Supporting Information). RT‐qPCR and IHC analyses confirmed the absence of CBP/p300 in the livers of AAV‐CRE mice (Figure S3B,C, Supporting Information). Consistent with the results observed in CBP/p300^LivDKO^ mice, blood glucose levels remained low in AAV‐CRE mice following intraperitoneal injection of alanine or glutamine (Figure 4F,G). Similarly, the capability of amino acid‐driven gluconeogenesis was significantly impaired in hepatocytes from AAV‐CRE mice (Figure 4H), with decreased mRNA and protein levels of amino acid metabolism genes (Figure 4I,J). These findings reveal that the disruption of amino acid catabolism leads to hypoglycemia due to impaired hepatic gluconeogenesis in the absence of CBP/p300.

### 2.5 Decreased Gluconeogenesis in CBP/p300^LivDKO^ Mice Is Independent of Gluconeogenic Genes

The phosphorylation of CBP at Ser436 induced by insulin has been shown to disrupt its binding to CREB in hepatocytes, thereby repressing key gluconeogenic gene expressions and hepatic glucose production.^[^ ^40^ ^]^ Unexpectedly, *Pck1* and *G6pc* expressions showed no alterations in RNA‐seq results from the livers of CBP/p300‐deficient mice (Figure 3D), which was further validated by RT‐qPCR (Figure 4K). Moreover, we observed significant increases in the protein levels of FOXO1 and PGC1α, two master coregulators of hepatic gluconeogenic gene transcription,^[^ ^43^, ^44^ ^]^ in CBP/p300‐null livers (Figure 4L). Additionally, the phosphorylation level of CREB at Ser 133 was even higher in the knockout mice (Figure 4L), which was supposed to promote the recruitment of CBP to the promoters of *Pck1* and *G6pc*.^[^ ^45^ ^]^ However, the binding ability of CREB and FOXO1 to the promoters of *Pck1* and *G6pc* remained unchanged in the livers of CBP/p300‐deficient mice (Figure 4M), suggesting alternative compensatory mechanisms for the regulation of gluconeogenic gene expression.

Despite unchanged mRNA levels, the hepatic protein level of PEPCK was elevated in CBP/p300‐deleted mice (Figure 4K,L). Post‐translational modifications (PTMs) including acetylation are known to regulate PEPCK protein stability, with acetylation promoting protein destabilization.^[^ ^46^ ^]^ Unexpectedly, the acetylation of PEPCK was significantly increased in CBP/p300^LivDKO^ mice, while its ubiquitination level decreased (Figure 4N). These findings indicate that the reduced hepatic gluconeogenesis in CBP/p300‐deficient mice primarily results from downregulation of most enzymes involved in amino acid catabolism, rather than changes in gluconeogenic gene expression (Figure 4O).

### 2.6 Histone Acylations Act Cooperatively in Regulating the Expression of Amino Acid Metabolism Genes

**Figure 5:**
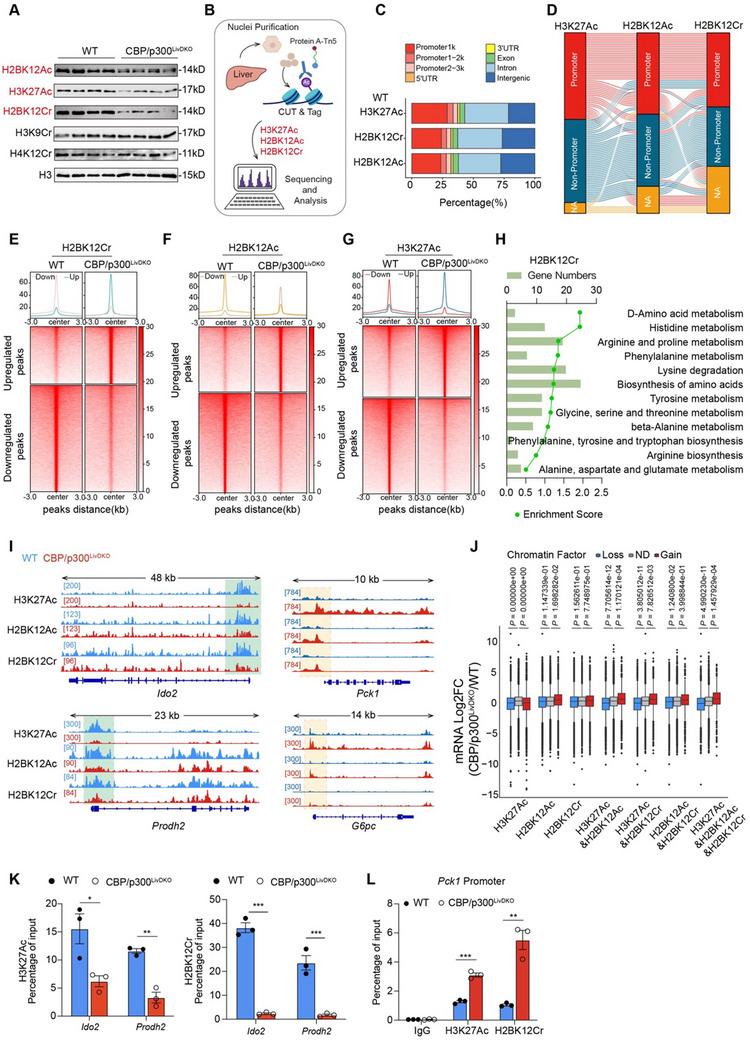
Histone acylations act cooperatively in regulating amino acid metabolism gene expression. A) Western blot analysis of site‐specific histone acylations in WT and CBP/p300^LivDKO^ mice (*n* = 4). Protein expression was normalized to total histone H3. B) Experimental design schematic depicting the assessment of H3K27Ac, H2BK12Ac, and H2BK12Cr histone marks in nuclei isolated from the livers of WT and CBP/p300^LivDKO^ mice using the CUT&Tag technique. C) Genome‐wide distribution of H3K27Ac, H2BK12Ac, and H2BK12Cr peaks in WT mouse livers. D) Sankey plot illustrating the relevance of H3K27Ac, H2BK12Ac, and H2BK12Cr distributions on amino acid metabolism genes. “NA: Not detected” indicates the absence of the histone modification on the gene. E–G) Heatmaps displaying the signal of specified histone modifications H2BK12Cr, H2BK12Ac, and H3K27Ac at peaks associated with differentially expressed genes in WT versus CBP/p300^LivDKO^ mice. H) KEGG analysis of the pathways showing decreased binding peaks of H2BK12Cr. I) Genome browser tracks of CUT&Tag signal at representative target gene loci. The green rectangles indicate significantly decreased peak regions of H3K27Ac, H2BK12Ac, and H2BK12Cr on target‐gene promoters. J) Fold‐change expression (CBP/p300^LivDKO^/WT; cut‐off: 1.5‐fold change) of genes that gain, lose, or have no difference (ND) in histone modifications H3K27Ac, H2BK12Ac, and H2BK12Cr (cut‐off: twofold change). K,L) ChIP‐qPCR analysis of the promoters of two amino acid metabolism genes and *Pck1* was performed using antibodies against H3K27Ac and H2BK12Cr in the livers from 6‐h fasted WT and CBP/p300^LivDKO^ mice (*n* = 3). Data are presented as mean ± SEM. Statistical significance was determined using two‐tailed unpaired Student's test: ^*^ *p* < 0.05, ^**^ *p* < 0.01, and ^***^ *p* < 0.001.

Recently, CBP/p300 have been demonstrated to utilize various acyl‐CoAs for histone acylations.^[^ ^22^, ^30^, ^31^ ^]^ Amino acid catabolism generates multiple acyl‐CoAs, such as acetyl‐CoA and crotonyl‐CoA,^[^ ^26^ ^]^ which serve as substrates for CBP/p300‐mediated histone modifications, thereby regulating gene expression. In the livers of CBP/p300^LivDKO^ mice, western blot analysis of acid‐extracted histones showed significant decreases in the levels of H2BK12Ac, H3K27Ac, and H2BK12Cr compared to WT controls (**Figure** **5**A). To explore the functional significance of these three modifications associated with CBP/p300 in controlling amino acid metabolism, we performed genome‐wide CUT&Tag^[^ ^47^ ^]^ analysis to identify candidate genes regulated by these modifications in livers (Figure 5B).

We first evaluated the distributions of these three histone modifications in WT mice. Surprisingly, H2BK12Cr, H3K27Ac, and H2BK12Ac showed broadly similar peak distributions (Figure 5C). There existed strong correlations among genome‐wide peaks for these three marks, particularly between H2BK12Cr and H2BK12Ac (Figure S4A, Supporting Information). A total of 15 221 target genes were enriched with all three modifications and classified into distinct KEGG pathways, including several amino acid metabolism pathways (Figure S4B,C, Supporting Information). Subsequently, we examined the distributions of these three marks on amino acid metabolism genes, identifying 50 amino acid metabolism genes linked with all three modifications (Figure S4D, Supporting Information). The Sankey plot depicted remarkably similar genomic distribution profiles of these modifications in WT mice (Figure 5D).

By integrating CUT&Tag profiles with RNA sequencing data, we unveiled a clear enrichment of H2BK12Cr, H2BK12Ac, and H3K27Ac peaks in downregulated genes of livers from CBP/p300^LivDKO^ mice compared to WT mice (Figure 5E–G). The downregulated peak genes were implicated in diverse amino acid metabolism pathways (Figure 5H and Figure S4E,F, Supporting Information). Specifically, the identified peaks highlighted candidate genomic loci at several amino acid metabolism genes including *Ido2* and *Prodh2*, revealing decreased levels of three marks at the promoters of these genes (Figure 5I). Significantly, the relative changes in histone acylations, in any pairwise combination at gene promoters, exhibited stronger correlations with changes in gene expression (Figure 5J). Chromatin immunoprecipitation‐quantitative PCR (ChIP‐qPCR) analysis validated significant decreases of H3K27Ac and H2BK12Cr levels at the promoters of *Ido2* and *Prodh2* in knockout mice compared with WT mice (Figure 5K).

Interestingly, the levels of H3K27Ac, H2BK12Ac, and H2BK12Cr marks at the promoters of gluconeogenic genes (*Pck1* and *G6pc*) showed notable increases in knockout mice (Figure 5I,L). Given these significant histone modification alterations, we examined the deacetylase family to assess deacetylase‐dependent regulation of *Pck1* and *G6pc* expressions. Downregulated *Hdac11* was a potential contributor to heightened histone marks at *Pck1* and *G6pc* promoters (Figure S5A, Supporting Information). To address this, hepatocytes isolated from wild‐type mice were transfected with an *Hdac11*‐overexpressing plasmid (OE‐HDAC11), which led to a substantial impairment of gluconeogenesis (Figure S5B, Supporting Information). Additionally, the downregulations of *Pck1* mRNA and protein levels were also validated in *Hdac11*‐overexpressed hepatocytes (Figure S5C,D, Supporting Information). *Hdac11* overexpression significantly enhanced its occupancy on the promoter of *Pck1* (Figure S5E, Supporting Information). These results indicate a crucial role for CBP/p300 in linking amino acid metabolism to gene expression through histone modifications.

### 2.7 The 2‐AAA/GCDH/H2BK12Cr Axis Promotes Hepatic Expression of Amino Acid Metabolism Genes in a Positive Feedback Loop

**Figure 6:**
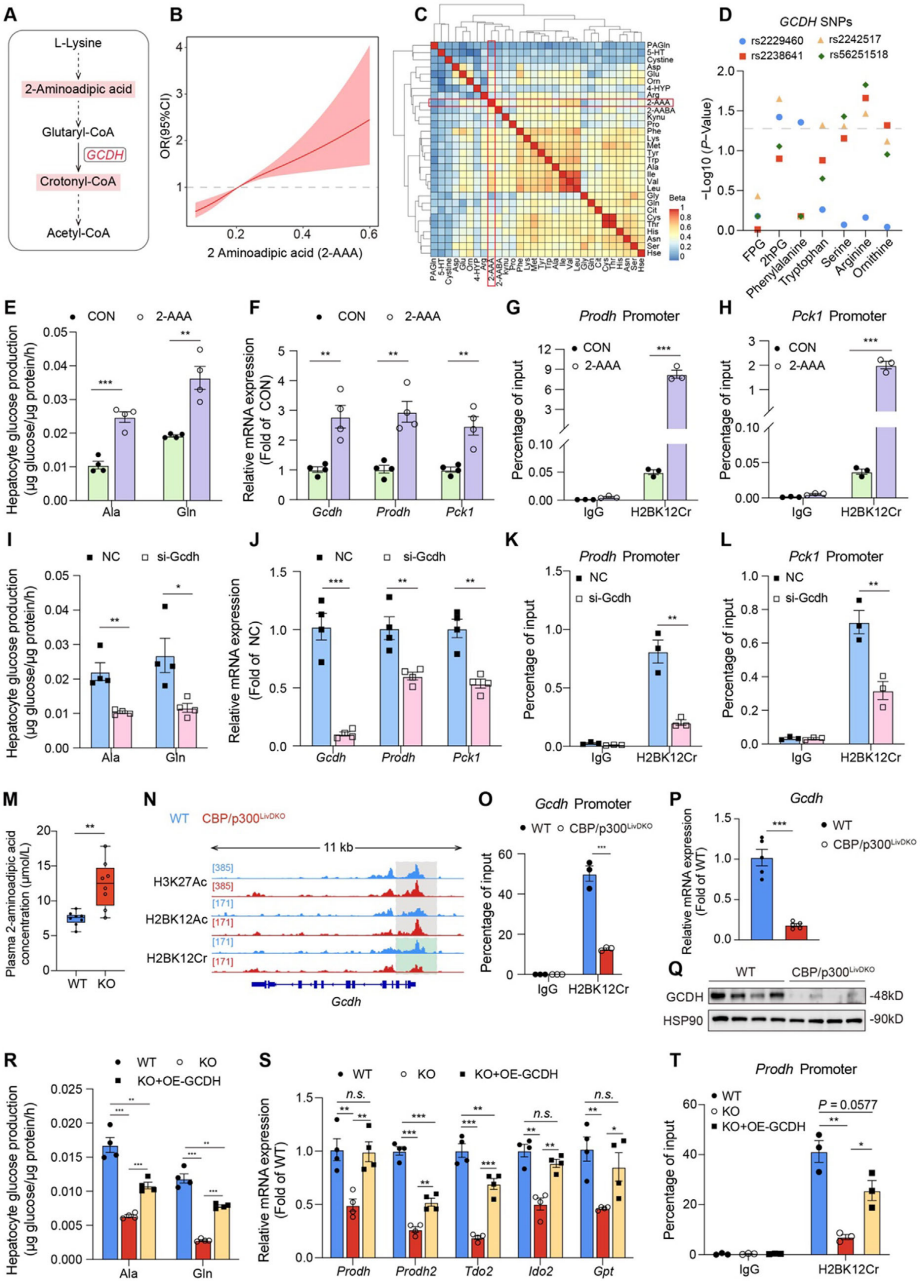
The 2‐AAA/GCDH/H2BK12Cr axis promotes hepatic expression of amino acid metabolism genes in a positive feedback loop. A) The 2‐AAA catabolism pathway. B) Restricted cubic spline (RCS) analysis depicting the association between circulating 2‐aminoadipic acid (2‐AAA) and incident type 2 diabetes. Gray dotted line represents an odds ratio (OR) of 1, while the pink shaded area signifies the 95% confidence interval (CI) of the OR. C) Spearman's correlation analysis of the association of circulating 2‐AAA with amino acids. Color key represents the regression coefficients of the independent variables. D) Correlations of four SNPs in human *GCDH* loci with fasting plasma glucose (FPG), 2‐h postprandial glucose (2h‐PG), and several circulating amino acids in the cohort. E,F) Glucose production driven by alanine and glutamine and related gene expressions in primary hepatocytes treated with or without 100 µM 2‐AAA (*n* = 4). G,H) ChIP‐qPCR analysis of *Prodh* and *Pck1* promoters using antibodies against H2BK12Cr in primary hepatocytes treated with or without 100 µM 2‐AAA (*n* = 3). I,J) Glucose production driven by alanine and glutamine and related gene expressions in normal control (NC) and si‐*Gcdh*‐treated hepatocytes (*n* = 4). K,L) ChIP‐qPCR analysis of *Prodh* and *Pck1* promoters using antibodies against H2BK12Cr in NC and si‐*Gcdh*‐treated hepatocytes (*n* = 3). M) Concentration of 2‐AAA in the plasma of WT and CBP/p300^LivDKO^ mice (*n* = 8). N) Genome browser tracks of CUT&Tag signal at the *Gcdh* gene loci. Green rectangle indicates significantly decreased peak regions of H2BK12Cr on *Gcdh* promoter. O) ChIP‐qPCR analysis of *Gcdh* promoter using antibodies against H2BK12Cr in the livers from 6‐h fasted WT and CBP/p300^LivDKO^ mice (*n* = 3). P,Q) *Gcdh* mRNA (*n* = 5) and protein (*n* = 4) expressions in the livers of WT and CBP/p300^LivDKO^ mice. R,S) Glucose production driven by alanine and glutamine and amino acid metabolism gene expressions in primary hepatocytes isolated from WT and CBP/p300^LivDKO^ mice (KO), followed by transfection with NC or overexpressing (OE)‐GCDH plasmids (*n* = 4). T) ChIP‐qPCR analysis of *Prodh* promoter using antibody against H2BK12Cr in WT, KO, and KO+OE‐GCDH hepatocytes (*n* = 3). Data are presented as mean ± SEM. Statistical significance was determined using two‐tailed unpaired Student's *t*‐test (E–P) or one‐way ANOVA, followed by Fisher's LSD test (R–T): ^*^ *p* < 0.05, ^**^ *p* < 0.01, and ^***^ *p* < 0.001; N.S., not significant.

2‐AAA derived from lysine degradation undergoes conversion into crotonyl‐CoA by GCDH (**Figure** **6**A), which may provide substrates for histone crotonylation modifications. We further investigated whether 2‐AAA and GCDH participated in CBP/p300‐governed cellular metabolism through histone crotonylation mechanisms. Multivariate‐adjusted restricted cubic spline (RCS) analysis demonstrated a strong association between elevated circulating 2‐AAA levels and increased risk of incident type 2 diabetes in Chinese adults (Figure 6B), corroborating findings from other human cohort studies.^[^ ^18^, ^19^ ^]^ Spearman correlation analysis revealed that circulating 2‐AAA level was correlated with various plasma amino acid concentrations (Figure 6C). Furthermore, association analysis identified significant correlations of specific SNPs in the human *GCDH* gene locus with fasting plasma glucose (FPG), 2‐h postprandial plasma glucose (2h‐PG), and several amino acids (Figure 6D). To validate the effects of 2‐AAA on glucose metabolism, primary mouse hepatocytes were treated with 100 µm 2‐AAA. Surprisingly, 2‐AAA treatment significantly enhanced gluconeogenesis driven by alanine, glutamine, and lactate/pyruvate (Figures 6E and S6A, Supporting Information). Consistently, 2‐AAA treatment substantially upregulated the expressions of *Gcdh*, *Prodh*, and *Pck1* (Figure 6F). Mechanically, the levels of H2BK12Cr at the promoters of *Prodh* and *Pck1* were markedly elevated following 2‐AAA treatment (Figure 6G,H).

To determine whether *Gcdh* regulates amino acid‐driven gluconeogenesis, primary hepatocytes were transfected with control or *Gcdh* siRNA. Knockdown of *Gcdh* significantly impaired hepatic gluconeogenesis derived from alanine, glutamine, and lactate/pyruvate (Figures 6I and S6B, Supporting Information), concomitant with decreased expression of *Prodh* and *Pck1* genes (Figure 6J). ChIP‐qPCR analysis confirmed that H2BK12Cr levels at the promoters of *Prodh* and *Pck1* were significantly reduced in *Gcdh*‐silenced hepatocytes (Figure 6K,L). Collectively, these results demonstrate that *Gcdh* plays a crucial role in regulating amino acid‐driven gluconeogenesis by providing substrates for histone crotonylation at the promoters of target genes.

Interestingly, we observed a significant increase in 2‐AAA levels in both plasma (Figure 6M) and liver (Figure S6C, Supporting Information) of CBP/p300^LivDKO^ mice, which seems to be paradoxical to reduced expression of amino acid metabolism genes and impaired amino acid‐driven gluconeogenesis in these mice. We hypothesize that decreased *Gcdh* expression in knockout mice may leads to 2‐AAA accumulation and consequent downregulation of amino acid metabolism genes. Supporting this hypothesis, H2BK12Cr level was decreased at the promoter of *Gcdh* in the livers of CBP/p300^LivDKO^ mice, while H3K27Ac and H2BK12Ac levels remained unchanged (Figure 6N). ChIP‐qPCR analysis validated these results (Figure 6O). Consistently, both mRNA and protein levels of *Gcdh* were significantly reduced in the livers of knockout mice (Figure 6P,Q and Figure S6D,E, Supporting Information). Importantly, overexpression of *Gcdh* in CBP/p300‐deficient hepatocytes partially restored the capacity for gluconeogenesis driven by alanine and glutamine (Figure 6R), reinstated the expression of several amino acid metabolism genes (Figure 6S), and replenished the level of H2BK12Cr at the promoter of *Prodh* (Figure 6T). Together, these results support existence of a 2‐AAA/GCDH/H2BK12Cr positive feedback loop that functions as a cell‐autonomous mechanism for metabolic rewiring in response to fluctuations of amino acid levels.

### 2.8 Targeting Hepatic Knockdown of *Crebbp*/*Ep300* Potently Attenuates Amino Acid‐Driven Gluconeogenesis in Diabetic Mice

**Figure 7:**
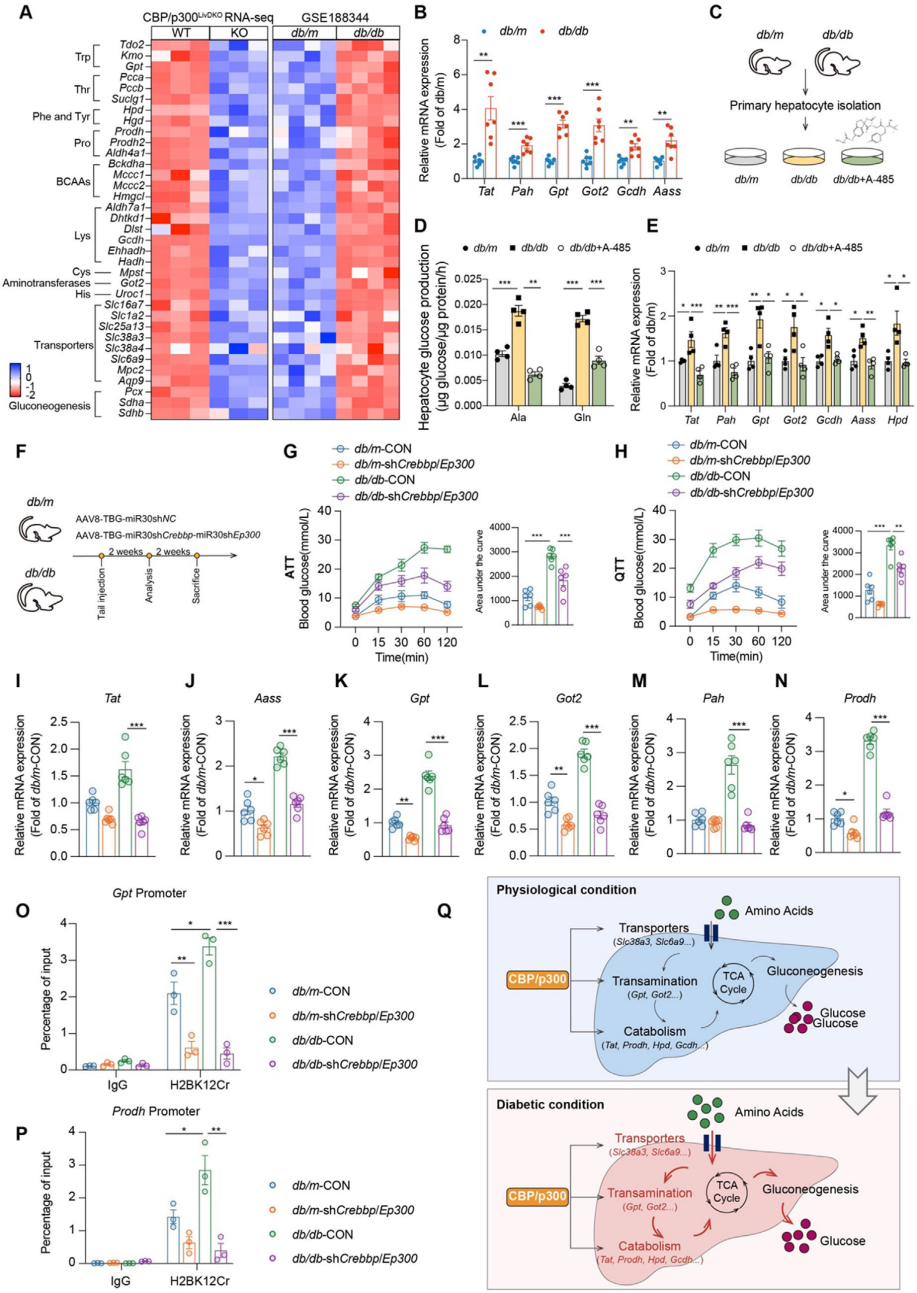
Hepatic amino acid metabolism is upregulated in diabetic mice. A) Heatmap displaying the relative expression of selected amino acid metabolism‐related genes. Left: RNA‐seq data from CBP/p300^LivDKO^ versus WT mice; Right: RNA‐seq data from GSE188344. B) RT‐qPCR analysis of the relative expression of amino acid metabolism genes in the livers of *db/m* versus *db/db* mice (*n* = 7). C) Experimental design schematic showing primary hepatocytes isolation from *db/m* and *db/db* mice, followed by treatment of *db/db* hepatocytes with 3 µM A‐485. D) Glucose production driven by alanine and glutamine in primary hepatocytes isolated from *db/m* and *db/db* mice treated with or without 3 µM A‐485(*n* = 4). E) RT‐qPCR analysis of the relative expression of amino acid metabolism genes in primary hepatocytes isolated from *db/m* and *db/db* mice treated with or without 3 µM A‐485 (*n* = 4). F) Experimental timeline showing the injection of AAV‐TBG‐miR30sh*NC* or AAV‐TBG‐miR30sh*Crebbp*‐miR30sh*Ep300* and subsequent analysis in *db/m* and *db/db* mice. G,H) Alanine or glutamine tolerance tests were performed on *db/m* and *db/db* mice following *Crebbp*/*Ep300* knockdown (*n* = 6 per group). Blood glucose levels were measured at indicated time points, and area under the curve (AUC) was calculated. I–N) mRNA expression of key amino acid metabolic genes in liver tissues from the four groups (*n* = 6 per group). O,P) ChIP‐qPCR analysis of H2BK12Cr enrichment of *Gpt* and *Prodh* promoters (*n* = 3 per group). Q) Schematic diagram illustrates the role of CBP/p300 in linking amino acid catabolism to hepatic glucose production by governing various aspects of amino acid metabolism. This regulatory loop is amplified under diabetic conditions. Disruption of this loop through CBP/p300 knockout or inhibition may serve as a therapeutic strategy to maintain amino acid and glucose homeostasis. Data are presented as mean ± SEM. Statistical analysis was performed using two‐tailed unpaired Student's *t*‐test (B) or one‐way ANOVA, followed by Fisher's LSD test (D,E,G–P): ^*^ *p* < 0.05, ^**^ *p* < 0.01, and ^***^ *p* < 0.001.

To further explore the changes in amino acid metabolism associated with diabetes, we analyzed RNA‐seq data from the livers of *db/db* mice obtained from the Gene Expression Omnibus (GEO) database. Several amino acid metabolism genes that were downregulated in CBP/p300^LivDKO^ mice showed significant upregulation in *db/db* mice (**Figure** **7**A). We validated these findings by showing the increased expressions of several amino acid metabolism genes, including *Tat*, *Pah*, *Gpt*, *Got2*, *Gcdh*, and *Aass* in the liver of *db/db* mice (Figure 7B). To examine the role of CBP/p300 under diabetic conditions, primary hepatocytes were isolated from *db/db* mice and treated with 3 µM A‐485, a selective CBP/p300 HAT inhibitor (Figure 7C). CBP/p300 inhibition by A‐485 significantly suppressed amino acid‐derived gluconeogenesis and reduced expression of amino acid metabolism genes in hepatocytes from *db/db* mice (Figure 7D,E).

We performed AAV‐mediated dual knockdown of *Crebbp*/*Ep300* using an AAV8‐TBG‐miR30sh*Crebbp*‐miR30sh*Ep300* construct in both *db/m* and *db/db* mice to investigate their roles in hepatic amino acid and glucose metabolism (Figure 7F). Notably, *db/db* mice exhibited significantly elevated blood glucose excursions following administration of either alanine or glutamine compared to *db/m* controls, demonstrating enhanced amino acid‐driven gluconeogenic capacity in the diabetic state (Figure 7G,H). Remarkably, *db/db* mice treated with sh*Crebbp*/*Ep300* showed markedly attenuated hepatic glucose production in response to both amino acids (Figure 7G,H). We subsequently examined the expressions of key amino acid metabolism genes in the liver tissues from these mice. *Tat*, *Aass*, *Gpt*, *Got2*, *Pah*, and *Prodh* expressions were significantly increased in *db/db* mice compared to *db/m* controls, which were effectively suppressed by hepatic *Crebbp/Ep300* knockdown (Figure 7I–N). ChIP‐qPCR analysis revealed that elevated H2BK12Cr enrichment at the promoters of *Gpt* and *Prodh* in *db/db* mice was substantially reduced by sh*Crebbp*/*Ep300* treatment (Figure 7O,P), providing compelling mechanistic evidence that CBP/p300‐mediated histone crotonylation directly governs the transcription of these key amino acid genes.

Collectively, these results demonstrate that upregulated hepatic amino acid metabolism represents a major contributor to hyperglycemia in type 2 diabetes, in which CBP/p300 may play a pivotal role (Figure 7Q).

## 3 Discussion

Certain amino acids and their metabolites are tightly linked to the development of type 2 diabetes.^[^ ^5^, ^48^ ^]^ However, the underlying mechanism how these amino acids, particularly ketogenic amino acids, modulate glucose homeostasis remain largely unexplored. In this study, liver‐specific ablation of CBP/p300 led to hyperaminoacidemia and hypoglycemia due to impaired amino acid catabolism and gluconeogenesis in the liver of mice. Numerous genes related to amino acid catabolism were downregulated in CBP/p300‐deficient livers, with decreased histone acetylation and crotonylation at their promoters. Interestingly, plasma and hepatic levels of 2‐AAA derived from lysine catabolism were elevated while its downstream catalyzing enzyme *Gcdh* was downregulated in knockout mice. 2‐AAA treatment or *Gcdh* overexpression increased hepatic glucose production by facilitating histone crotonylation modification at the promoters of target genes. Knockdown of *Gcdh* showed an opposite result. Furthermore, targeted hepatic knockdown of *Crebbp*/*Ep300* attenuated amino acid‐induced gluconeogenesis, effectively lowering blood glucose in *db/db* mice. Genetic variants in human *CREBBP/EP300* and *GCDH* genes correlated with circulating amino acid and glucose levels. These findings partially explain why 2‐AAA and ketogenic amino acids are strongly associated with the risk of type 2 diabetes.

CBP/p300 function as acetyltransferases and transcriptional coactivators that regulate hepatic energy homeostasis.^[^ ^32^ ^]^ Under fasting conditions, glucagon promotes CREB phosphorylation through cAMP‐PKA pathway, leading to recruitment of CBP/p300 to CRE‐containing genes including *Pck1* and *G6pc*.^[^ ^49^, ^50^ ^]^ CBP or p300 knockdown decreases expressions of the two genes and impairs gluconeogenesis.^[^ ^39^, ^40^, ^51^ ^]^ Under high glucose and insulin conditions, p300 acetylates ChREBP on Lys672 and increases its transcriptional activity, facilitating its recruitment to the promoters of lipogenic target genes.^[^ ^41^ ^]^ CBP/p300 also acetylate the critical lipogenic activator SREBP1c and increases its stability, leading to hepatic lipid accumulation.^[^ ^52^ ^]^ These studies indicate a dominant role for CBP/p300 in maintaining glucose and lipid homeostasis. In the present study, liver‐specific deletion of CBP/p300 in mice led to the elevation of circulating and hepatic amino acid levels, accompanied by hypoglycemia and impaired gluconeogenesis. These findings identify CBP/p300 as crucial coordinators of hepatic amino acid and glucose metabolism.

Previous studies have established CBP/p300 as transcriptional regulators of key gluconeogenic genes.^[^ ^39^, ^40^, ^51^ ^]^ Besides CREB, the transcription factor FOXO1 also binds to the promoters of *Pck1* and *G6pc* to regulate their expressions during fasting.^[^ ^44^, ^53^, ^54^ ^]^ Unexpectedly, the transcription of *Pck1* and *G6pc* remained unaltered in CBP/p300‐deficient livers, despite the increased levels of FOXO1 protein expression and CREB phosphorylation. In addition to transcription factors, core histone tail modifications such as acetylation and crotonylation are essential for the decompaction of chromatin fibers, thereby influencing gene expressions.^[^ ^55^ ^]^ Importantly, permissive chromatin, characterized by its dynamic and open state, allows transcription factors to bind and initiate gene expression.^[^ ^56^ ^]^ Our CUT&Tag analysis revealed elevated levels of H3K27Ac, H2BK12Ac, and H2BK12Cr modifications at the promoters of *Pck1* and *G6pc* in CBP/p300‐deficent liver, indicating a potential compensatory role of histone deacetylases. Indeed, histone deacetylase HDAC11 was dramatically downregulated in CBP/p300^LivDKO^ mice, and its overexpression suppressed hepatic gluconeogenesis alongside reduced expressions of *Pck1* and *G6pc*. It is likely that increased chromatin accessibility due to downregulated HDAC11 and uncompromised transcriptional activity of CREB and FOXO1 likely ensures continuous expression of gluconeogenic genes in CBP/p300‐deficient livers. This regulatory mechanism may serve as an essential protective strategy to prevent lethal hypoglycemia following CBP/p300 loss.

Amino acid breakdown produces ammonium (NH_4_^+^) and a carbon skeleton. The carbon skeleton of amino acids can be converted into TCA cycle intermediates, which are further utilized to generate ATP or provide precursors for gluconeogenesis and fatty acid synthesis. Excess NH_4_^+^ is converted to urea by the liver and excreted by the kidney.^[^ ^1^ ^]^ Generally, the urea cycle is intricately linked to amino acid‐based gluconeogenesis.^[^ ^57^ ^]^ Early studies demonstrated that glucagon infusion resulted in sharp decreases in plasma amino acid levels, while glucagon deficiency exhibited opposite effects.^[^ ^58^, ^59^, ^60^ ^]^ Knockout of glucagon gene or its receptor, as well as glucagon receptor antagonism, elevated plasma and hepatic levels of amino acids concomitant with reduced urea production due to downregulated urea cycle enzymes.^[^ ^61^, ^62^, ^63^ ^]^ Disruption of glucagon signaling in humans with non‐functioning glucagon receptors led to marked hyperaminoacidemia.^[^ ^64^ ^]^ Conversely, individuals with glucagonomas experienced profound hypoaminoacidemia and increased urea synthesis upon amino acid infusion.^[^ ^65^, ^66^ ^]^ In essence, the interplay between ammonia detoxification and amino acid‐derived gluconeogenesis is harmoniously regulated by glucagon. However, CBP/p300‐null livers displayed the decoupling of carbon skeletons and nitrogen from amino acid catabolism, with decreased gluconeogenesis and increased ureagenesis. We observed notable increases in histone acyl modifications at the promoters of *Cps1*, *Ass1*, *Arg1*, and *Asl* in CBP/p300^LivDKO^ mice (data not shown) and their expressions, suggesting a compensatory mechanism for ureagenesis. Further exploration revealed a significant upregulation of some genes involved in ammonium production, such as *Sds*, *Mat1a*, *Aass*, *Asns*, *Gls*, and *Got1* in CBP/p300^LivDKO^ mice (Figure 3H). Since substrate availability is the primary determinant of the ureagenesis rate,^[^ ^67^ ^]^ we interpret the upregulation of several ammonium‐producing genes as indicative of the body's need to mobilize amino acid catabolism for energy support. Amino acid deamination is crucial for initiating amino acid catabolism. Whereas, the α‐keto acids generated from amino acid deamination are unable to undergo further catabolism in CBP/p300 knockout mice, necessitating urgent ammonium detoxification through substrate‐driven ureagenesis.

Adaptation to varying nutrient availability is essential for maintaining metabolic balance within cells and organisms.^[^ ^68^, ^69^ ^]^ Acyl‐CoAs, derived from several metabolic pathways including amino acid catabolism, lipid metabolism, and ketone body metabolism, play crucial roles in linking nutritional signal to gene expressions through post‐translational protein modifications. Lysine and tryptophan catabolism generate acetyl‐CoA and crotonyl‐CoA, which provide substrates for histone acetylation and crotonylation modifications.^[^ ^26^ ^]^ Recent research has shown that lysine catabolism was reprogrammed in glioblastoma stem cells, leading to intracellular crotonyl‐CoAs accumulation and histone H4 lysine crotonylation.^[^ ^28^ ^]^ As both histone crotonyltransferase and histone acetyltransferase, CBP/p300 have been demonstrated to stimulate gene transcription by regulating histone lysine acetylation and crotonylation.^[^ ^30^, ^70^ ^]^ In this study, CUT&Tag and RNA sequencing datasets revealed significant associations of H2BK12Ac, H3K27Ac, and H2BK12Cr modifications with the expression of genes. The three histone modifications displayed obvious decreases at the promoters of various amino acid metabolism genes downregulated in the liver of CBP/p300^LivDKO^ mice. Despite potential competition between acetylation and crotonylation at lysine residues like H2BK12, CBP/p300 deficiency blocks histone acylation modifications at target genes as the writer of both acylation types, and impaired amino acid catabolism further exacerbates this effect due to substrates shortage required for acylations. Therefore, no compensatory increase occurs in one modification when another type of acylation is erased. Prior research has highlighted the indispensable role of hormone signaling in amino acid homeostasis.^[^ ^71^ ^]^ Our findings uncover a novel cell‐autonomous mechanism that delineates the crucial role of amino acids in maintaining their own homeostasis.

2‐AAA derived from lysine catabolism has been identified as a biomarker of diabetes risk in three European cohorts.^[^ ^19^ ^]^ We have replicated this observation among Chinese adults. However, the underlying mechanisms remain largely unknown. Puzzlingly, 2‐AAA intervention led to reduced fasting plasma glucose levels in mice fed both standard chow and high‐fat diets, with increased fasting plasma insulin levels. 2‐AAA treatment stimulated insulin secretion from BTC6 cells as well as isolated mouse and human islets.^[^ ^19^ ^]^ Another study revealed that elevated levels of 2‐AAA correlated with obesity and impaired insulin signaling.^[^ ^72^ ^]^ In this study, 2‐AAA treatment significantly promoted gluconeogenesis in primary mouse hepatocytes involved in increased expressions of *Pck1* and amino acid metabolism genes through H2BK12Cr modification. Elevated circulating level of lysine has been demonstrated to associate with the risk of developing type 2 diabetes.^[^ ^11^ ^]^ It is possible that its metabolite 2‐AAA links this ketogenic amino acid to glucose homeostasis through histone crotonylation‐mediated hepatic gluconeogenesis.

In cells, acetyl‐CoA is the predominant CoA species, and crotonyl‐CoA is about 1000‐fold less abundant than acetyl‐CoA.^[^ ^30^ ^]^ Given the extremely low intracellular level and inherent instability of crotonyl‐CoA, the dynamic change of histone crotonylation is more sensitive to crotonyl‐CoA level fluctuation.^[^ ^28^ ^]^ GCDH is the key enzyme for the conversion of 2‐AAA into crotonyl‐CoA. Interestingly, genetic sequencing analysis revealed associations between SNPs within *GCDH* gene loci and plasma glucose as well as amino acid levels among Chinese adults. 2‐AAA significantly induced *Gcdh* expression (Figure 6F), suggesting a potential feedback regulation. Moreover, overexpression of GCDH in CBP/p300^LivDKO^ hepatocytes increased H2BK12Cr modification and expression of amino acid metabolism genes (Figure 6S,T), indicating that GCDH might regulate histone crotonylation through a CBP/p300‐independent mechanism. Although we cannot exclude the involvement of additional histone crotonyltransferases, it is reasonable to suppose that GCDH‐regulated histone crotonylation predominantly operates through a substrate‐driven mechanism. This notion is supported by Liu et al., demonstrating that increased crotonyl‐CoAs robustly promoted histone crotonylation on native calf thymus histones in vitro, even without any enzymatic catalysis.^[^ ^73^ ^]^ Thus, substrate availability alone could influence histone crotonylation levels. Additionally, our data indicate a dual role for CBP/p300 within this regulatory network. On one hand, CBP/p300 function as histone acyltransferases that directly regulate histone acylations; on the other hand, they also modulate *Gcdh* expression, thereby controlling crotonyl‐CoA production and influencing substrate availability. Hence, even a slight uptick in the flux of 2‐AAA in hepatocytes will lead to enhanced signaling for amino acid metabolism.

Previous reports demonstrated that histone acetyltransferase (HAT) activity of p300 was enhanced in the livers of obese and type 2 diabetic mice. p300 overexpression exacerbated glucose intolerance and insulin resistance, with increased hepatic expression of PEPCK and G6Pase.^[^ ^41^ ^]^ Our study revealed redundant effects of CBP and p300 on glucose and amino acid metabolism. Only dual hepatic knockout of these two genes decreased circulating amino acid and glucose levels, without significant changes even in triallelic deletion of CBP/p300. We further investigated the role of hepatic CBP/p300 in modulating amino acid and glucose metabolism in diabetic mice. Through in vivo AAV‐mediated dual knockdown of *Crebbp*/*Ep300* in *db/db* mice, we observed significant reductions in blood glucose levels during alanine and glutamine tolerance tests, indicating effective suppression of amino acid‐driven gluconeogenesis. *Crebbp*/*Ep300* knockdown normalized the aberrantly elevated expression of key amino acid metabolism genes in the livers of *db/db* mice, with reduced H2BK12Cr enrichment at these gene promoters. Our study identifies CBP/p300‐mediated histone crotonylation as a critical epigenetic mechanism underlying dysregulated hepatic amino acid metabolism associated with diabetes.

In summary, our findings firmly establish CBP/p300 as key regulators linking amino acid metabolism to glucose homeostasis. Hepatic deletion of CBP/p300 reprogrammed amino acid metabolism, with downregulation of carbon skeleton catabolism and upregulation of nitrogen metabolism. The 2‐AAA/GCDH axis was implicated in CBP/p300‐governed expressions of amino acid metabolism genes by promoting histone crotonylation, ultimately boosting amino acid‐driven gluconeogenesis. This novel cell‐autonomous model reveals the link between amino acid catabolism and gene expressions via histone crotonylations, accounting for the causative role of 2‐AAA in the development of type 2 diabetes. Interventions targeting 2‐AAA/GCDH regulatory loop or CBP/p300‐mediated histone crotonylation represent promising strategies for the treatment of type 2 diabetes.

## 4 Limitations of the Study

Several limitations should be acknowledged in this study. First, we observed decoupling of carbon skeletons and nitrogen from amino acid catabolism in CBP/p300^LivDKO^ mice, which is inconsistent with the well‐established effects of glucagon signal disruption. The underlying mechanisms need to be explored. Second, hepatic in vivo knockout of CBP/p300 failed to inhibit the transcription of *Pck1* and *G6pc*. Beyond HDAC11, other histone acylation erasers may be also involved in the transcription of gluconeogenic genes. Third, we did not investigate the acylation levels and activities of nonhistone proteins under the condition of CBP/p300 ablation. Finally, beyond the molecular mechanisms elucidated in this study, other amino acids may modulate metabolic signaling pathways through the generation of distinct substrates, such as propionyl‐CoA and succinyl‐CoA. Future investigations will be required to explore the roles of alternative acylation modifications in CBP/p300‐governed amino acid metabolism.

## 5 Experimental Section

### Human Study

The study protocol received approval from the Institutional Review Board of Ruijin Hospital affiliated to the Shanghai Jiao Tong University School of Medicine (Approval No. 2011‐14), and written informed consent was obtained from all participants. Study samples used in this study were derived from the China Cardiometabolic Disease and Cancer Cohort (4C) Study, a nationwide, population‐based, prospective cohort study with up to 5 years follow‐up.^[^ ^7^ ^]^ In this study, 1707 incident diabetes cases were included from the 4C cohort, matched with 1707 individuals with normal glucose regulation (NGR) individuals at baseline using propensity score matching (PSM). The logistic model incorporated age, gender, body mass index (BMI), and FPG. Sociodemographic characteristics (sex and age) and lifestyle factors (smoking status and alcohol intake) were collected. Other clinical parameters including BMI, systolic blood pressure (SBP), triglyceride (TG), total cholesterol (TC), low‐density lipoprotein cholesterol (LDL‐C), and high‐density lipoprotein cholesterol (HDL‐C) were collected as previously described.^[^ ^7^ ^]^ All participants underwent measurements for blood glucose (FPG, 2h‐PG, and HbA1c) and homeostatic model assessment of insulin resistance (HOMA‐IR). Blood samples were collected and immediately centrifuged at 4 °C, followed by separation and storage at‐−80 °C. Fasting plasma samples were analyzed for amino acids using the UPLC‐MS/MS methods. DNA samples for SNP array genotyping underwent quality assessment and control procedures using PLINK following established protocols.^[^ ^74^ ^]^ Imputation was performed with IMPUTE2 using the 1000G Phase3 panel according to the pipeline described by Elisabeth et al.^[^ ^75^ ^]^ Following quality control, 3004 samples and 7693929 SNPs or InDels were retained for genotype‐phenotype association analysis. Detailed information is provided in Supplementary data online, Table S1 (Supporting Information).

### Mice

Mice were housed under a 12‐h day‐night cycle with ad libitum access to water and standard chow in a specific‐pathogen‐free facility (20 ± 2 °C ambient temperature; 40–70% humidity). *Crebbp^flox/flox^* and *Ep300^flox/flox^* mice were purchased from the GemPharmatech Co. Ltd (Nanjing, China). *Alb*‐Cre mice were purchased from the Jackson Laboratory (Bar Harbor, Maine, USA). Male *db/db* and their lean *db/m* littermates (aged 4 and 8 weeks) were purchased from Beijing Vital River Laboratory Animal Technology Co., Ltd (Beijing, China). In general, *Crebbp^flox/flox^/Ep300^flox/flox^* mice, which were generated by crossing floxed *Crebbp* mice and floxed *Ep300* mice, were mated with *Alb*‐Cre mice. The resulting progeny (The F1 generation) *Crebbp* ^flox/wt^/*Ep300* ^flox/wt^; *Alb*‐cre mice were further intercrossed with *Crebbp* ^flox/wt^/*Ep300* ^flox/wt^ mice to generate *Crebbp* ^flox/flox^/*Ep300* ^wt/wt^; *Alb*‐cre (CBP^LivKO^) mice, *Crebbp* ^wt/wt^/*Ep300* ^flox/flox^; *Alb*‐cre (p300^LivKO^) mice*, Crebbp* ^flox/flox^/*Ep300* ^flox/wt^; *Alb*‐cre (CBP^LivKO^/p300^HET^) mice, *Crebbp* ^flox/wt^/*Ep300* ^flox/flox^; *Alb*‐cre (CBP^HET^/p300^LivKO^) mice and *Crebbp* ^flox/flox^/*Ep300* ^flox/flox^; *Alb*‐cre (CBP/p300^LivDKO^) mice. Cre‐negative littermates from each breeding setup served as wild‐type (WT) control. All mice were kept on C57BL/6J background. Animal protocols were approved by the Institutional Animal Care and Use Committee of Shanghai Model Organisms Center, Inc., Shanghai, China (Approval No. 2019‐0026).

### Genomic PCR for Mouse Genotyping

Mice genotype was performed using genomic DNA extracted from tail tissue with the One Step Mouse Genotyping Kit (Vazyme, PD101‐01). Two pairs of primers were used to identify *Crebbp/Ep300* floxed alleles: *Crebbp*‐F1/*Crebbp*‐R1 and *Ep300*‐F1/*Ep300*‐R1 (sequences provided in Supporting Information). The *Crebbp*‐F1 and *Crebbp*‐R1 primers were used to distinguish WT (147 bp) or floxed *Crebbp* allele (245 bp), whereas the *Ep300*‐F1 and *Ep300*‐R1 primers were used to distinguish WT (416 bp) or floxed *Ep300* allele (521 bp). *Alb*‐Cre‐F and *Alb*‐Cre‐R primers were used to detect the *Alb*‐Cre allele (450 bp for *Alb*‐Cre^+/‐^ and none for Alb‐Cre^−/−^). The primer sequences used for genotyping mice are provided in Table S2 (Supporting Information).

### AAV Virus Preparation and In Vivo Transduction

For in vivo hepatocyte‐specific deletion or knockdown of *Crebbp*/*Ep300*, adeno‐associated viral vectors serotype 8 (AAV8; OBiO Technology, Shanghai) driven by the hepatocyte‐specific thyroxine‐binding globulin (TBG) promoter were administered via tail vein injection at a dose of 5 × 10^11^ genome copies per mouse. Sixteen‐week‐old *Crebbp^flox/flox^*/*Ep300^flox/flox^* mice received AAV8 expressing Cre recombinase (AAV8‐TBG‐Cre) or a control vector without transgene, followed by 6‐h fasting before blood and tissue collection. Additionally, 4‐week‐old male *db/db* and littermate *db/m* mice were injected with AAV8 encoding miR30‐based shRNAs targeting both *Crebbp* and *Ep300* (AAV8‐TBG‐miR30sh*Crebbp*‐miR30sh*Ep300*) or non‐targeting scrambled shRNA control (AAV8‐TBG‐shNC), followed by 16‐h fasting prior to sample collection. Tissues were rapidly harvested, immediately frozen in liquid nitrogen, and stored at −80 °C until further analysis.

### Metabolic Phenotyping

Basal blood glucose levels were measured using a Glucocard glucometer after 6‐h fasting. Alanine tolerance test (ATT) and glutamine tolerance test (QTT) were conducted by intraperitoneal injection of L‐alanine or L‐glutamine (Sigma‐Aldrich), respectively, followed by serial measurements of blood glucose levels. Specifically, *Crebbp*/*Ep300* knockout mice received injections at doses of 2 g kg^−1^ L‐alanine or 1 g kg^−1^ L‐glutamine, whereas *db/db* and *db/m* mice were administered doses of 1 g kg^−1^ L‐alanine or 0.5 g kg^−1^ L‐glutamine. Area under the curve (AUC) values were calculated to quantify glucose responses.

### Plasma Chemistry Assays

*Total amino acid quantification*: Total amino acid levels were quantified using a Total Amino Acid assay kit (Nanjing Jiancheng Bioengineering Institute) according to the manufacturer's instructions.

*Urea assay*: Urea concentrations in plasma and primary hepatocyte cultures were measured using a Urea Assay Kit (Nanjing Jiancheng Bioengineering Institute), following the manufacturer's protocol.

*Triglycerides (TG) and total cholesterol (TC) assays*: Plasma TG and TC levels were determined using commercially available assay kits (Shanghai Kehua Bio‐engineering Co., Ltd.), according to the manufacture's guidelines.

*Non‐esterified fatty acids (NEFAs) assay*: Plasma NEFA levels were quantified using a NEFA Assay Kit (FUJIFILM Wako Pure Chemical Corporation), following the manufacturer's instructions.

### Primary Hepatocyte Isolation

Primary hepatocytes were isolated and purified using the two‐step collagenase perfusion method.^[^ ^76^ ^]^ Briefly, mice were anesthetized and fixed on a surgery pad. A “U”‐shaped incision exposed the liver, which was perfused with buffers to facilitate cell isolation. Following gentle dissection and filtration, the cell suspension was centrifuged. Non‐parenchymal cells and dead hepatocytes were removed, leaving viable hepatocytes that were resuspended in Hepatocyte Medium (ScienCell Research Laboratories, #5201) for subsequent experiments.

### Cell Transfection

Primary hepatocytes were transfected with mouse *Gcdh* siRNA (OBiO Technology, Shanghai) or plasmid CV702‐3xFlag‐*Gcdh* (Genechem, Shanghai, China) using Lipofectamine 2000 transfection reagent, according to the manufacturer's instruction. The complementary DNA target sequences of siRNA are provided in Table S3 (Supporting Information).

### Glucose Production by Primary Hepatocytes

Primary hepatocytes were pre‐treated with 100 nM dexamethasone (Sigma‐Aldrich) for 16 h. The medium was then replaced with glucose‐production buffer consisting of glucose‐free DMEM (Gibco, A1443001) supplemented with 10 mm L‐glutamine or 10 mM L‐alanine in the presence of 100 µM 8‐Bromoadenosine 3′,5′‐cyclic monophosphate (Sigma‐Aldrich). After 24‐h incubation, medium was collected to quantify glucose levels using a colorimetric glucose assay kit (Applygen), following the manufacturer's instructions. The results were normalized to the protein content.

### Immunohistochemistry (IHC) and Immunofluorescence (IF) Staining

For IHC, harvested liver tissues were fixed in 4% paraformaldehyde and embedded in paraffin. Sections of 2‐µm thickness were deparaffinized, rehydrated, and subjected to antigen retrieval in antigen‐unmasking solution (Vector Laboratories, H‐3300) using a slow cooker at 120 °C for 10 min. Afterward, the sections were incubated overnight at 4 °C with primary antibodies against CBP (Cell Signaling Technology, #7289, 1:100 dilution) and p300 (Cell Signaling Technology, #86377, 1:100 dilution) diluted in Dako Antibody Diluent (Agilent, Dako, S302283). Next, HRP‐conjugated secondary antibodies were applied for 1 h at room temperature, followed by DAB Substrate Kit (Cell Signaling Technology, #13079) development for ≈10 min. The sections were counterstained with hematoxylin and dehydrated with ethanol and xylene prior to mounting.

For IF, isolated primary hepatocytes were plated on Millicell EZ Slide 8‐well glass (Millipore, PEZGS0896), and subject to fixation with 4% paraformaldehyde. Samples were permeabilized with PBS containing 0.5% Triton X‐100. Alexa Fluor 488 and 594 (YEASEN, 33706ES60, and 33112ES60, 1:400 dilution) were used as secondary antibodies. Nuclei were stained with 4,6‐diamidino‐2‐phenylindole (DAPI) (SouthernBiotech, 0100‐20).

IHC slides were imaged on a StrataFAXSPLUS S (TissueGnostics) and IF slides were imaged using a Zeiss LSM 880 microscope.

### Protein Isolation and Western Blot Analysis

Total proteins were isolated from snap‐frozen liver tissues by homogenization in lysis buffer containing protease and phosphatase inhibitors (MedChemExpress, HY‐K0010/HY‐K0021/HY‐K0022). Samples were centrifugated for clarification, and protein concentration was determined using the BCA Protein Assay Kit (Thermo Scientific, 23227). For cultured cells, lysates were prepared in Laemmli Sample Buffer (Bio‐Rad, 1610737), boiled, and resolved on SDS‐PAGE gels. Proteins were transferred onto PVDF membranes (Millipore, IPVH00010), blocked, and incubated with primary antibodies overnight. Following washing with TBST, membranes were incubated with HRP‐conjugated secondary antibodies and visualized using a LAS‐4000 Super CCD Remote Control Science Imaging System (Fuji). The primary antibodies used in the experiments are provided in Table S4 (Supporting Information).

### Co‐Immunoprecipitation (Co‐IP)

Liver tissues were harvested and homogenized in the lysis buffer. Lysates were incubated with PCK1 antibody (Proteintech, 16754‐1‐AP, 1:100 dilution) or normal IgG for 4 h and then with protein A/G‐magnetic beads overnight at 4 °C. The immunoprecipitated proteins were washed and eluted with SDS loading buffer. Then standard western blotting was followed.

### Histone Extraction and Purification

The histones from liver tissue or primary hepatocytes were extracted and purified using an EpiQuik Total Histone Extraction Kit (Epigentek, OP‐0006‐100) according to the manufacturer's instructions.

### RNA Extraction and RT‐qPCR

Total RNA was extracted from tissues or cells using Trizol regent (Thermo Scientific, 15596026CN), and quantified by a NanoDrop ND2000 spectrophotometer (Thermo Scientific). One microgram of RNA was transcribed to cDNA with HiScript III RT SuperMix for qPCR with gDNA wiper (Vazyme, R323‐01). RT‐qPCR was performed using ChamQ Universal SYBR qPCR Master Mix (Vazyme, Q711‐03) in an Applied Biosystems 7300 Real‐Time PCR machine (Applied Biosystems). *18s* was used as the internal control. The primer sequences used for RT‐qPCR were listed in Table S5 (Supporting Information).

### RNA‐seq Library Preparation, Sequencing, and Analysis

The library construction and sequencing were performed at Shanghai Sinomics Corporation (Shanghai, China). Total RNA was extracted using RNeasy mini kit (Qiagen) from mouse livers. One microgram of total RNA per sample was used to prepare the sequencing library by mRNA‐seq Lib Prep Kit for Illumina (ABclonal, China) following Sample Preparation Guide. Sequencing was performed on Illumina NovaSeq 6000 (Illumina, USA). After quality control, raw sequencing data was pretreated into trimmed data and further compared with *Mus musculus* genome by using Hisat2 software. The differentially expressed genes and transcripts (measured by fragments per kilobase of exon per million reads mapped (FPKM) value) were identified by setting a threshold at fold change ≥1.5, *p*‐value < 0.05.

### Metabolomics Analysis

Metabolomics analysis was performed using the Q300 Kit (Metabo‐Profile, Shanghai, China). Briefly, standards for all targeted metabolites obtained from Sigma‐Aldrich (St. Louis, MO, USA), Steraloids Inc. (Newport, RI, USA), and TRC Chemicals (Toronto, Ontario, Canada) were accurately weighed and prepared at a concentration of 5.0 mg mL^−1^. After derivatization, the mouse livers and plasma samples (*n* = 8 per group) were transferred to a new 96‐well plate with 10 µL of internal standards in each well. An ultraperformance liquid chromatography coupled to tandem mass spectrometry (UPLC‐MS/MS) system (ACQUITY UPLC‐Xevo TQ‐S, Waters Corp., Milford, MA, USA) was used to quantify all targeted metabolites. Three types of quality control samples (i.e., test mixtures, internal standards, and pooled biological samples) were used. The raw data files generated by UPLC‐MS/MS were processed using MassLynx software (v4.1, Waters, Milford, MA, USA) to perform peak integration, calibration, and quantitation. Metabolites with VIP (Variable importance in projection, obtained based on the OPLS‐DA model) ≥1.0 and *p*‐value <0.05 (univariate analyses were based on whether the data were normally distributed) were regarded as statistically significant.

### CUT&Tag Assays

CUT&Tag assays were performed by Jiayin Biotechnology Ltd. (Shanghai, China). Briefly, native nuclei were purified from frozen liver tissues of WT and CBP/p300^LivDKO^ mice as previously described.^[^ ^77^ ^]^ 5 × 10^5^ nuclei were washed twice gently with wash buffer (20 mM HEPES, pH 7.5; 150 mM NaCl; 0.5 mM Spermidine; 1× Protease inhibitor cocktail). 10 µL concanavalin A‐coated magnetic beads (Bangs Laboratories) were added per sample and incubated at room temperature for 10 min. Bead‐bounded cells were then suspended with dig wash buffer (20 mM HEPES, pH 7.5; 150 mM NaCl; 0.5 mM Spermidine; 1× Protease inhibitor cocktail; 0.05% Digitonin; and 2 mM EDTA). Samples were incubated overnight at 4 °C with rotation using primary antibodies against H3K27Ac (Cell Signaling Technology, #8173, 1:100 dilution), H2BK12Ac (PTM BIO, PTM‐108, 1:100 dilution), H2BK12Cr (PTM BIO, PTM‐509, 1:100 dilution) or appropriate normal IgG (Millipore, 12‐370, 1:50 dilution) to establish background levels and ensure specificity. After removal of primary antibody using magnet strand, cells were incubated with secondary antibody (Millipore, AP132, 1:100 dilution) for 1 h and then incubated with pA‐Tn5 adapter complex for 1 h. After a wash with Dig‐med buffer, cells were resuspended in Tagmentation buffer (10 mM MgCl_2_ in Dig‐med buffer) and incubated at 37 °C for 1 h. Genomic DNA was isolated using phenol‐chloroform‐isoamyl alcohol extraction and ethanol precipitation. Sequencing libraries were prepared according to the manufacturer's instructions and cleaned up using XP beads (Beckman Counter). Sequencing was performed in the Illumina NovaSeq 6000 using PE150. After quality control, the reads were mapped to *Mus musculus* genome using the BWA program. Peak calling was performed using MACS2 software with optimized parameters tailored specifically for each histone modification based on their distinct signal profiles, employing a stringent threshold (*q*‐value < 0.05). Peaks were annotated with the ChIPseeker package. Differential peaks were determined by setting thresholds of fold change ≥2 and *p*‐value < 0.05. Coverage, reads, and peaks were visualized with the Integrative Genomics Viewer (IGV).

### Chromatin Immunoprecipitation (ChIP) Assays

The ChIP assay was performed using an EZ‐ChIP Chromatin Immunoprecipitation kit (Millipore, 17‐371) according to the manufacturer's protocol. Briefly, liver tissues or primary hepatocytes were immediately cross‐linked in 1% formaldehyde for 15 min, which was then stopped by glycine and homogenized in cell lysis buffer. Then the samples were sheared to 100–1000 bp by ultrasonic treatment. The sheared chromatin was incubated with antibodies for H3K27Ac, H2BK12Cr, CREB, FOXO1, His‐Tag, or normal IgG. The precipitated chromosome was then pulled down, purified, and quantified by RT‐qPCR. Primers used for ChIP‐qPCR assays were listed in Table S6 (Supporting Information).

### Statistical Analysis

For the human genetic study, genotype‐phenotype association tests were performed using PLINK2 with gender and age as covariates. Associations between SNPs within the genes (*CREBBP*, *EP300*, and *GCDH*) and plasma amino acids and glucose levels were assessed using linear regression. The relationship between log‐transformed circulating 2‐AAA and type 2 diabetes incidence was modeled using restricted cubic splines with three knots placed at the 5th, 50th, and 95th percentiles. The model was fully adjusted for age, sex, BMI, smoking status, alcohol intake, education attainment, family history of diabetes, SBP, FPG, TC, LDL‐C, and HDL‐C. Spearman correlation analysis was used to assess correlations between 2‐AAA and other amino acids. All experiments were replicated at least three times for each condition. Statistical analyses were performed using GraphPad Prism 9.0. Two‐tailed Student's *t*‐test was used for comparisons between two groups, and one‐way ANOVA followed by Fisher's LSD post hoc test was used for comparisons among three or more groups. Statistical significance was declared at *p* < 0.05. Significance levels are indicated as: ^*^ *p* < 0.05; ^**^ *p* < 0.01; ^***^ *p* < 0.001. All data were presented as means ± SEM (standard error of the mean).

### Ethics approval statement

The human study protocol was approved by the Institutional Review Board of Ruijin Hospital affiliated to the Shanghai Jiao Tong University School of Medicine and informed written consent was obtained from study participants (Approval No. 2011‐14). All animal experiments were conducted according to the ethical policies and procedures approved by the Institutional Animal Care and Use Committee of Shanghai Model Organisms Center, Inc., Shanghai, China (Approval No. 2019‐0026).

## Conflict of Interest

The authors declare no conflict of interest.

## Supporting information



Supporting Information

Supporting Information

Supporting Information

## Data Availability

The *Crebbp* ^flox/flox^ and *Ep300* ^flox/flox^ mice are available upon request. Further information and requests for resources and reagents should be directed to and will be fulfilled by the Lead Contact, Libin Zhou (zlb11178@rjh.com.cn). Source data are provided with this paper. The data that support the findings of this study are available from the corresponding author upon reasonable request.
