# Supplementary material for: Hepatic CBP/p300 Orchestrate Amino Acid‐Driven Gluconeogenesis through Histone Crotonylation
Source: Adv Sci (Weinh). 2025 Aug 12;12(41):e07635. doi: 10.1002/advs.202507635 (PMC12591208; doi:10.1002/advs.202507635)
Supplement: Supplementary file 1 — Supporting Information [file ADVS-12-e07635-s001.docx]

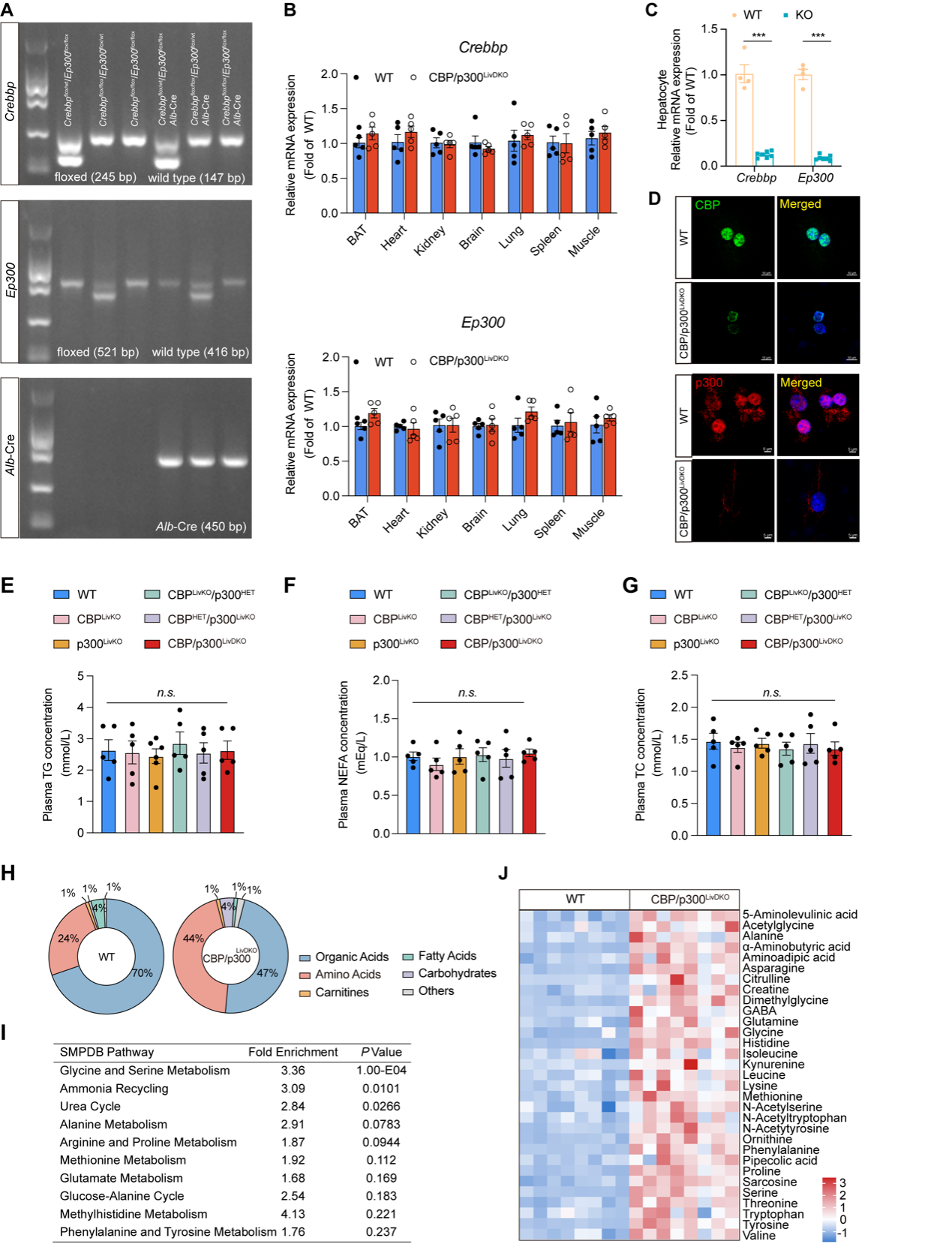


**Figure S1.** Validation of liver-specific deletion of *Crebbp* and *Ep300* in CBP/p300^LivDKO^ mice. **A)** Genomic PCR characterization of tail samples from 1-week-old mice of the indicated genotype. The top and middle lanes show the *Crebbp* and *Ep300* floxed alleles. The bottom lane displays the presence of *Alb-Cre*. **B)** Relative expression of *Crebbp* and *Ep300* in non-hepatic tissues of WT and CBP/p300^LivDKO^ mice by RT-qPCR (*n* = 5). **C)** RT-qPCR analysis of the *Crebbp* and *Ep300* mRNA expression in primary hepatocytes isolated from WT and CBP/p300^LivDKO^ mice (*n* = 4). **D)** Immunostaining of CBP (green), p300 (red) and nuclei (blue) in primary hepatocytes isolated from WT and CBP/p300^LivDKO^ mice. Scale bar = 10 μm. **E-G)** Plasma triglycerides (TG), free fatty acids (NEFAs), and cholesterol (TC) concentration in 6-hour fasted mice from six groups (*n* = 5 per group). **H)** Donut plot showing the distribution of metabolite classes in WT and CBP/p300^LivDKO^ mice. Key metabolite classes are highlighted. **I)** Metabolite set enrichment analysis using SMPDB (Small Molecule Pathway Database). **J)** Heatmap of statistically altered metabolites involved in amino acid metabolism. Rows show the *Z* scores calculated for each group. Data are presented as mean ± SEM. Statistical significance was determined using two-tailed unpaired Student’s t-test **(B and C)** or one-way ANOVA followed by Fisher’s LSD test **(E-G)**, compared with WT group: ^***^*P* < 0.001. *n.s.*, not significant.


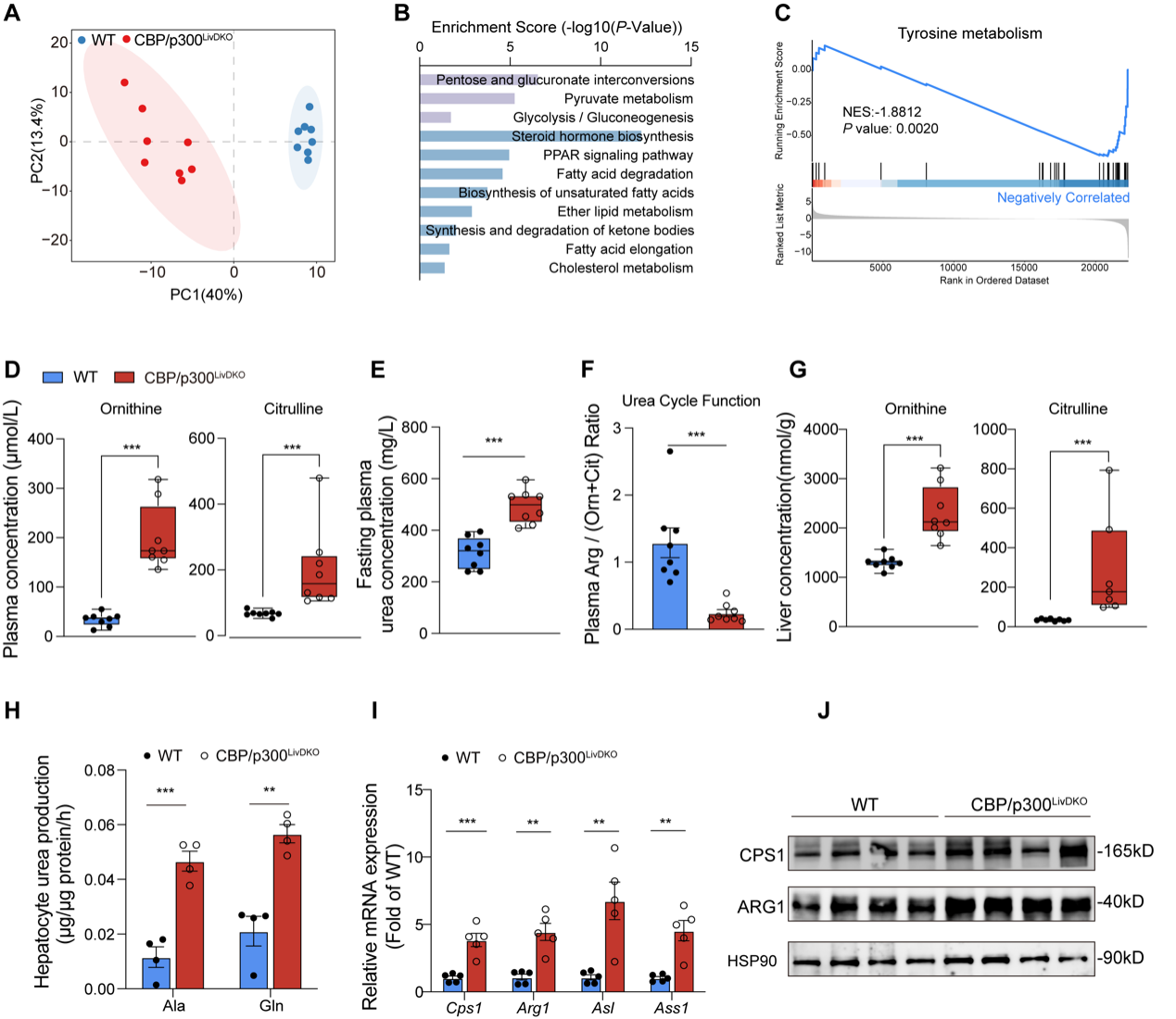


**Figure S2.** Urea cycle metabolites and urea production are paradoxically upregulated in CBP/p300^LivDKO^ mice. **A)** PCA score plot illustrating distinct metabolic signatures in the livers of WT and CBP/p300^LivDKO^ mice (*n* = 8). **B)** KEGG Pathway enrichment analysis of RNA-seq data from WT and CBP/p300^LivDKO^ mice liver. Pathways are clustered with similar colors based on their overall function. **C)** GSEA plots of tyrosine metabolism pathway in RNA-seq data. NES, normalized enrichment score. **D)** Concentrations of ornithine and citrulline in the plasma of WT and CBP/p300^LivDKO^ mice (*n* = 8). **E)** Concentration of urea in the plasma of WT and CBP/p300^LivDKO^ mice (*n* = 8). **F)** Urea cycle function reflected by the ratio of plasma arginine: (ornithine + citrulline) in CBP/p300^LivDKO^ *versus* WT mice (*n* = 8). **G)** Concentrations of ornithine and citrulline in the liver tissues of WT and CBP/p300^LivDKO^ mice (*n* = 8). **H)** Urea production in primary hepatocytes isolated from WT and CBP/p300^LivDKO^ mice (*n* =3). **I)** RT-qPCR analysis of mRNA expression of urea cycle-related genes in the liver tissues of 6-hour fasted WT and CBP/p300^LivDKO^ mice (*n* = 5). **J)** Western blot analysis of urea cycle enzymes in the liver tissues of WT and CBP/p300^LivDKO^ mice (*n* = 4). Data are presented as mean ± SEM. Statistical significance was determined using two-tailed unpaired Student’s t-test: ^**^*P* < 0.01, and ^***^*P* < 0.001.


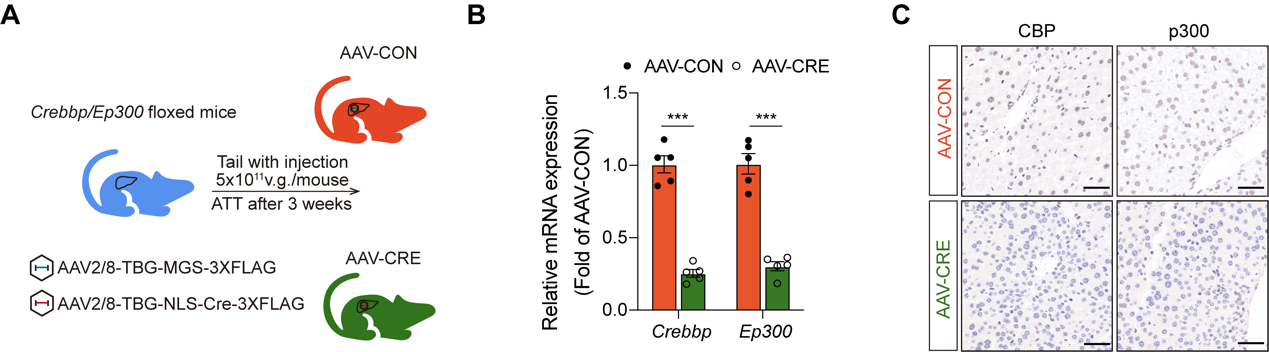


**Figure S3****.** Generation and validation of adult hepatic *Crebbp/Ep300* double knockout mice. **A)** Schematic diagram showing the experimental design. **B)** Quantitation of *Crebbp* and *Ep300* mRNA expression in the liver tissues of AAV-CON and AAV-CRE mice via RT-qPCR (*n* = 5). **C)** Immunohistochemistry staining of CBP and p300 in AAV-CON and AAV-CRE mouse livers. Scale bar = 100 μm. Data are presented as mean ± SEM. Statistical analysis was performed using two-tailed unpaired Student’s t-test: ^***^*P* < 0.001.

**
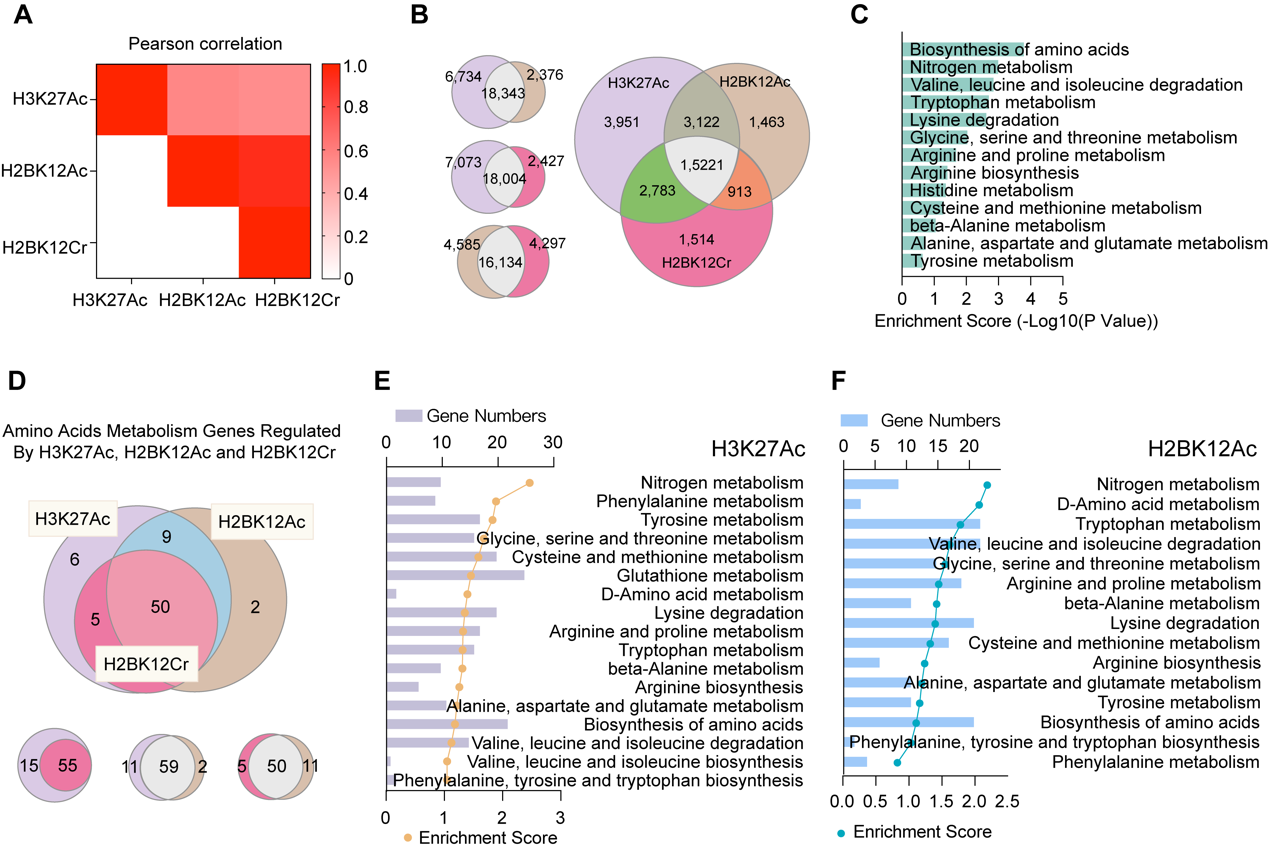
**

**Figure S4.** The overview of H2BK12Cr, H2BK12Ac, and H3K27Ac peaks distribution. **A)** Pearson correlation of genome-wide signal difference among H3K27Ac, H2BK12Ac, and H2BK12Cr. **B, C)** Venn diagram showing the overlap among three indicated histone marks peaks and KEGG analysis of the overlapped binding peaks at genes. **D)** Venn diagram showing the overlap among three indicated histone marks peaks covering amino acid genes. **E, F)** KEGG analysis of the pathways showing decreased binding peaks of H3K27Ac and H2BK12Ac.


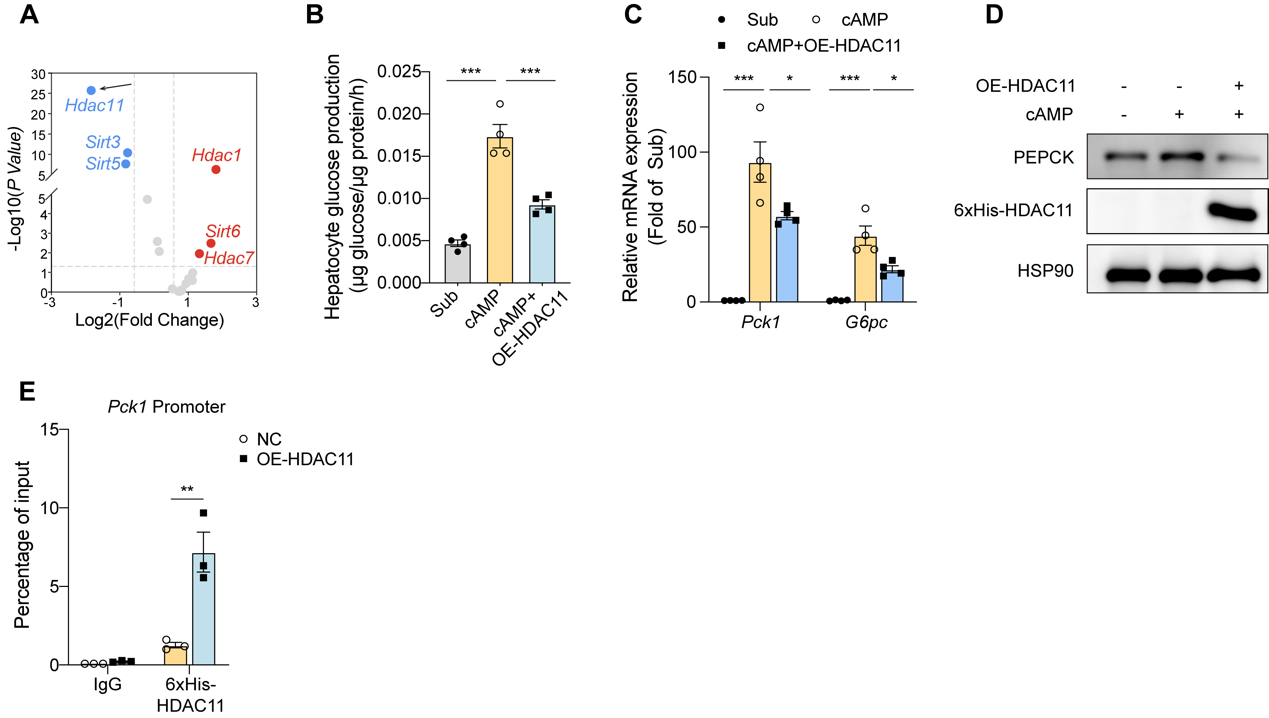


**Figure S5.** Overexpression of HDAC11 inhibits hepatocyte glucose production and gluconeogenic gene expression. **A)** Volcano plot showing differentially expressed deacetylase genes in 6-hour fasted WT and CBP/p300^LivDKO^ mice liver (*n* = 3). The two vertical lines indicate the cutoff points for a 1.5-fold change. The horizontal line indicates the *P* < 0.05 significance cutoff. **B)** Glucose production in primary hepatocytes isolated from wild-type mice, followed by transfection with NC or OE-HDAC11 plasmid (*n* = 4). **C)** The mRNA levels of gluconeogenic genes in primary hepatocytes transfected with NC or OE-GCDH plasmid (*n* = 4). **D)** Western blot analysis of PEPCK protein levels in primary hepatocytes transfected with NC or OE-GCDH plasmid (*n* = 3). **E)** ChIP-qPCR analysis of *Pck1* gene promoter using antibody against 6 His in hepatocytes transfected with NC or OE-GCDH plasmid (*n* = 3). Data are presented as mean ± SEM. Statistical analysis was conducted using two-tailed unpaired Student’s t- test (**E**) or one-way ANOVA, followed by Fisher’s LSD test (**B, C**): ^*^*P* < 0.05, ^**^*P* < 0.01, and ^***^*P* < 0.001.


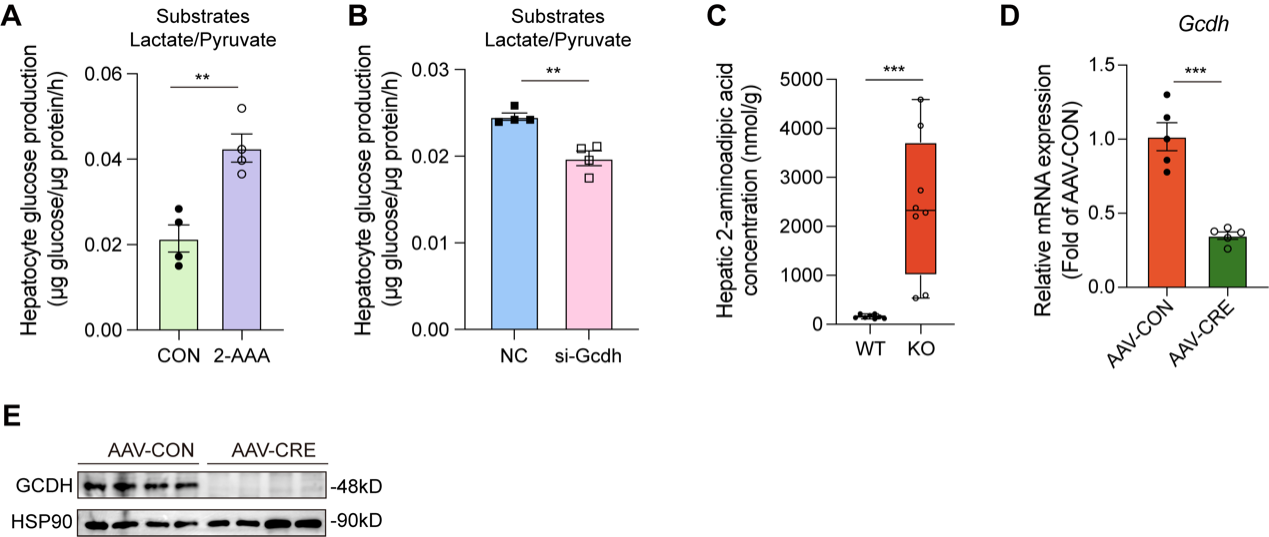


**Figure S6.** The 2-AAA/GCDH/H2BK12Cr axis fosters hepatic gluconeogenesis in a positive feedback loop. **A)** Glucose production driven by lactate/pyruvate in primary hepatocytes treated with or without 100 μM 2-AAA (*n* = 4). **B)** Glucose production driven by lactate/pyruvate in NC and si-*Gcdh* hepatocytes (*n* = 4). **C)** Concentration of 2-AAA in the livers of WT and CBP/p300^LivDKO^ mice (*n* = 8). **D)** Quantitation of *Gcdh* mRNA expression via RT-qPCR in the livers of AAV-CON and AAV-CRE mice (*n* = 5). **E)** Western blot analysis of GCDH in AAV-CON and AAV-CRE mice (*n* = 4). Data are presented as mean ± SEM. Statistical analysis was performed using two-tailed unpaired Student’s t test: ^**^*P* < 0.01, and ^***^*P* < 0.001.
