# Supplementary material for: Hepatic CBP/p300 Orchestrate Amino Acid‐Driven Gluconeogenesis through Histone Crotonylation
Source: Adv Sci (Weinh). 2025 Aug 12;12(41):e07635. doi: 10.1002/advs.202507635 (PMC12591208; doi:10.1002/advs.202507635)
Supplement: Supplementary file 2 — Supporting Information [file ADVS-12-e07635-s002.zip › 5-Table S1 workflow_v2.docx]

**Table S1, Supporting Information**

**Quality Control Process for Human Genetic Data**
