# Supplementary material for: Hepatic CBP/p300 Orchestrate Amino Acid‐Driven Gluconeogenesis through Histone Crotonylation
Source: Adv Sci (Weinh). 2025 Aug 12;12(41):e07635. doi: 10.1002/advs.202507635 (PMC12591208; doi:10.1002/advs.202507635)
Supplement: Supplementary file 3 — Supporting Information [file ADVS-12-e07635-s003.docx]

**Supplementary Tables**

Supplementary Table S1

Supplementary Table S2. The primer sequences used for genotyping mice

| Name | Species | Sequence (5’->3’) |
| --- | --- | --- |
| *Crebbp*-F1 | Mouse | GGTTGCCCAGAAGGGAATTGC |
| *Crebbp*-R1 | Mouse | GGAAAATGAAGAGTGCAGTGACTC |
| *Ep300*-F1 | Mouse | TGGTCAGGCTTTTGGAGCAAG |
| *Ep300*-R1 | Mouse | TAGCAACAGGCTCAGTGGTTACG |
| *Alb*-Cre-F | Mouse | TGGCAAACATACGCAAGGG |
| *Alb*-Cre-R | Mouse | CGGCAAACGGACAGAAGCA |

Supplementary Table S3. The cDNA target sequences of siRNA

| Name | Species | Sequence (5’->3’) |
| --- | --- | --- |
| Gcdh-mouse-498 Forward | Mouse | CGAGGAACAGCGACAGAAATT |
| Gcdh-mouse-498 Reverse | Mouse | UUUCUGUCGCUGUUCCUCGTT |

Supplementary Table S4.The primary antibodies used in Western blot, IHC, IF, IP, and ChIP-qPCR analysis

| Antibody | Source | Dilution | Identifier |
| --- | --- | --- | --- |
| Anti-CBP | CST | 1:100 (IHC/IF) | 7389 |
| Anti-p300 | CST | 1:100 (IHC/IF) | 86377 |
| Anti-HSP90 | CST | 1:1000 (WB) | 4877 |
| Anti-FOXO1 | CST | 1:1000 (WB), 1:50 (ChIP-qPCR) | 2880 |
| Anti-p-CREB (Ser133) | CST | 1:1000 (WB) | 9198 |
| Anti-CREB | CST | 1:1000 (WB) ,1:50 (ChIP-qPCR) | 9197 |
| Anti-Acetylated-Lysine | CST | 1:1000 (WB) | 9441 |
| Anti-H3K27Ac | CST | 1:1000 (WB), 1:100 (ChIP-qPCR) | 9173 |
| Anti-His-Tag | Proteintech | 1:1000 (WB),1:100 (ChIP-qPCR) | 66005-1-Ig |
| Anti-PCK1 | Proteintech | 1:1000 (WB), 1:100 (IP) | 16754-1-AP |
| Anti-BCKDHA | Santa Cruz | 1:500 (WB) | Sc-271538 |
| Anti-H3 | PTMBio | 1:1000 (WB) | PTM-6613 |
| Anti-H2BK12Ac | PTMBio | 1:1000 (WB) | PTM-153 |
| Anti-H2BK12Cr | PTMBio | 1:1000 (WB), 1:100 (ChIP-qPCR) | PTM-509 |
| Anti-H4K12Cr | PTMBio | 1:1000 (WB) | PTM-523 |
| Anti-H3K9Cr | PTMBio | 1:1000 (WB) | PTM-516RM |
| Anti-CPS1 | Abclonal | 1:1000 (WB) | A8080 |
| Anti-ARG1 | Abclonal | 1:1000 (WB) | A1847 |
| Anti-KYNU | Abclonal | 1:1000 (WB) | A19245 |
| Anti-GPT | Abclonal | 1:1000 (WB) | A23962 |
| Anti-TDO2 | Abclonal | 1:1000 (WB) | A6766 |
| Anti-PGC1α | Abclonal | 1:1000 (WB) | A12348 |
| Anti-GLUD1 | Abclonal | 1:1000 (WB) | A7631 |
| Anti-Ubiquitin | Abclonal | 1:1000 (WB) | A0162 |
| Anti-GCDH | Abclonal | 1:1000 (WB) | A9057 |

Supplementary Table S5. Primers sequences used for RT-qPCR

| Name | Species | Sequence (5’->3’) |
| --- | --- | --- |
| *18s* | Mouse | Forward: GCAATTATTCCCCATGAACG  Reverse: GGCCTCACTAAACCATCCAA |
| *Gapdh* | Mouse | Forward: ATTCAACGGCACAGTCAA  Reverse: CTTCTGGGTGGCAGTGAT |
| *Crebbp* | Mouse | Forward: AATGCCCTACCCTGCTCCA  Reverse: CCATGCTCTGTTTGCTGGCT |
| *Ep300* | Mouse | Forward: CCAAGCGGCCTAAACTCTCA  Reverse: AAGCTGACTGATATCGCCACC |
| *Cps1* | Mouse | Forward: GCCACACAGCTTTCTTTCACAC  Reverse: ATGTGTGCTGTCTTTGCCTTCAC |
| *Ass1* | Mouse | Forward: TCAAGGGCCGAAATGATCTGATG  Reverse: TATCTCAAGGACATCTGGGCTGT |
| *Asl* | Mouse | Forward: GCAGAAGCGGATCAATGTCTTG  Reverse: GGTCATACACAGAGAAGCCCAA |
| *Arg1* | Mouse | Forward: GACATCAACACTCCCCTGACAA  Reverse: CCAATCCCCAGCTTGTCTACTT |
| *Tdo2* | Mouse | Forward: GGCTGGAAAGAACACCTGGTT  Reverse: TGACAGTCGTCGTTCACCTTTAC |
| *Ido2* | Mouse | Forward: CTTAATCGAGAACCGCCAGC  Reverse: TCTTGGCAGCACCTTTTGGG |
| *Kynu* | Mouse | Forward: TAAGCCTACTCCAAAGCGGC  Reverse: AAGGTCTCTTCCCCCTCTCG |
| *Gpt* | Mouse | Forward: TTCAAGAAGGTGCTCACGGA  Reverse: CATCTGTTTCTGCACCTCGG |
| *Got2* | Mouse | Forward: ATGTCTGCCTCTGCCAATCG  Reverse: TTTCACCTCTTGCAACCATTGCT |
| *Cbs* | Mouse | Forward: CAAACAGCCTATGAGGTGGAAG  Reverse: GGAATCTTCATCGTTGCTCTTG |
| *Mpst* | Mouse | Forward: CAGTGAGTGGATGGGGTTTGG  Reverse: GCTCAGATTGCGGGGCTGTTT |
| *Gcat* | Mouse | Forward: CACCTGGAAGAGTGAGCGTG  Reverse: AGATTCTTATGGATGCTCTGGGTCC |
| *Hal* | Mouse | Forward: TATTGTGGCTGCCTTGACCC  Reverse: ACCTGTGGCTTTCTGCGATT |
| *Prodh2* | Mouse | Forward: GGCAACTAAGCGCATGTGGG  Reverse: TTCTCCTGGGCTCTTCGGAT |
| *Gcdh* | Mouse | Forward: ACCTATGGGAGCGAGGAACA  Reverse: CAGGGGAGTTGGTGATCCAGG |
| *Glud1* | Mouse | Forward: GTAATGTGGGCCTGCACTCT  Reverse: ACAGTCAGCCTCCAAGATGC |
| *Bckdha* | Mouse | Forward: GGCCGGATCTCCTTCTACAT  Reverse: CTTGCAGCCGTAGTGAACAG |
| *Slc6a9* | Mouse | Forward: TGTACATCTATGGGCACCGT  Reverse: TGGGATGCAGATGACAGACG |
| *Pck1* | Mouse | Forward: GTGCTGGAGTGGATGTTCGG  Reverse: CTGGCTGATTCTCTGTTTCAGG |
| *G6pc* | Mouse | Forward: ACTGTGGGCATCAATCTCCTC  Reverse: CGGGACAGACAGACGTTCAGC |
| *Hdac11* | Mouse | Forward: AAGGCATCTCCAGAGCCACCAT  Reverse: CAGGGTAGATGTGGCGGTTGTA |
| *Prodh* | Mouse | Forward: AGCTGGTACGTGGTGCATAC  Reverse: GGCCTATCTCCTTCATCCTGC |
| *Tat* | Mouse | Forward: ATTGGCTGAGTCTATGGGGATT  Reverse: TTCGTCGATCAGAGATTCCAGT |
| *Pah* | Mouse | Forward: CTGTCACAGAGTTCCAGCCTCT  Reverse: ACCCTTTGAGTGTAGGGGTCAT |
| *Aass* | Mouse | Forward: TGGCATTACAGTCATTGGTGAG  Reverse: CAGATAGCTAGCAGGCTGCATT |
| *Hpd* | Mouse | Forward: TCAGCCACGTCATCAAGCAA  Reverse: CGCAGTCTTCCACCTCGAAT |

Supplementary Table S6. Primers sequences used for ChIP-qPCR

| Name | Species | Sequence (5’->3’) |
| --- | --- | --- |
| CREB-*Pck1*-promoter | Mouse | Forward: GCTATGATCCAAAGGCCTGC  Reverse: GGAAGGCCAACTGTGCTTG |
| CREB-*G6pc*-promoter | Mouse | Forward: TTTGCTATTTTACGTAAATCACCCT  Reverse: GTACCTCAGGAAGCTGCCA |
| FOXO1-*Pck1*-promoter | Mouse | Forward: GTGGGAGTGACACCTCACAGC  Reverse: AGGGCAGGCCTAGCCGAGACG |
| FOXO1-*G6pc*-promoter | Mouse | Forward: GTGCCTGTTTTGCTATTTTACG  Reverse: GATTCAGTCTGTAGGTCAATCC |
| H2BK12Cr-*Ido2*-promoter | Mouse | Forward: GAACAGGTCTTGGCTATGC  Reverse: GCTGCTCCAAAGGTCAAGAC |
| H2BK12Cr-*Prodh2*-promoter | Mouse | Forward:CAGTGAGGTCTGGTATGAGGAG  Reverse: CTTTCTGAAATGGGTTGGTGGC |
| H2BK12Cr-*Prodh*-promoter | Mouse | Forward: GTCTCCATGGCCATCAGACTA  Reverse: GCCTTCGTTCAAGCTCATGAT |
| H2BK12Cr-*Gpt*-promoter | Mouse | Forward: CTGCCTTACCAACTCCCTCTG  Reverse: GGAGTCTGCGTGAGGTAAGG |
| H2BK12Cr-*Pck1*-promoter | Mouse | Forward: GCACTCTGTTTGGGATGGCT  Reverse: CATGGCCCACTTCCCTGTAG |
